# Offline Reinforcement Learning for Adaptive Support in AI-Assisted Decision-Making

**DOI:** 10.1145/3815418

**Published:** 2026-08-08

**Authors:** ZANA BUÇINCA, SIDDHARTH SWAROOP, AMANDA E. PALUCH, SUSAN A. MURPHY, KRZYSZTOF Z. GAJOS

**Affiliations:** Massachusetts Institute of Technology, Cambridge, Massachusetts, USA; University College London, London, UK; University of Massachusetts Amherst, Amherst, Massachusetts, USA; Harvard University, Boston, Massachusetts, USA; Harvard University, Boston, Massachusetts, USA

**Keywords:** AI-assisted decision-making, overreliance, human-centric objectives, human-centered AI, explainable AI, human-AI interaction, decision support systems

## Abstract

AI decision-support tools typically offer a fixed type of assistance, like AI recommendations and explanations, regardless of the specific decision, individual, or broader context. This fixed design has been shown to hinder both human-AI decision accuracy and human skill improvement in the task. We posit that AI assistance needs to be dynamic, changing in response to contextual factors (e.g., AI uncertainty, task difficulty), individual differences, and specified objectives (e.g., decision accuracy, skill improvement). To enable such adaptive support, we propose reinforcement learning (RL) as a general approach for modeling human-AI decision-making to optimize human-AI interaction for diverse objectives. RL enables optimizing various objectives in AI-assisted decision-making by tailoring and adaptively providing decision support to humans — the right type of assistance, to the right person, at the right time. We instantiated our approach with two objectives: human-AI accuracy on the decision-making task and human skill improvement (i.e., learning about the task) and learned decision support policies from previous human-AI interaction data. We compared the optimized policies against several baselines in AI-assisted decision-making. Across two experiments (N = 316 and N = 964), our results consistently demonstrated that people interacting with policies optimized for accuracy achieve significantly higher accuracy — and even human-AI complementarity — compared to those interacting with any other type of AI support. Our results further indicated that human learning was more difficult to optimize than accuracy. While the policies learned the best available actions to optimize learning, participants who interacted with learning-optimized policies showed significant learning improvement only at times. Our research (1) demonstrates offline RL to be a promising approach to model the dynamics of human-AI decision-making, leading to policies that may optimize various objectives and provide novel insights about the AI-assisted decision-making space, and (2) emphasizes the importance of considering skill improvement and other human-centric objectives beyond accuracy in AI-assisted decision-making, opening up the novel research challenge of optimizing human-AI interaction for such objectives.

## 1 INTRODUCTION

Research on adaptive interfaces has shown that personalizing interactions based on individual differences, devices, environments, or tasks can improve interaction efficiency, accessibility, and user acceptance of the interface [37, 43, 66, 75, 87, 108]. These findings reflect an important insight of human-computer interaction (HCI): *static, one-size-fits-all* systems often fail to meet the varied needs and goals of diverse users in different contexts. We argue that this well-studied principle in HCI, should also guide human–AI interaction, where AI systems must increasingly support diverse users, contexts, and interaction objectives.

Current AI-powered decision-support systems are typically non-adaptive, offering the same type of AI assistance to every individual, in each situation and for every decision. Most commonly, this assistance takes the form of what we refer to as a Simple Explainable AI (SXAI) interaction technique, where the AI provides a decision recommendation accompanied by an explanation intended to help decision-makers understand the reasoning behind the AI’s suggestion and assess its quality. While the SXAI technique is intuitively appealing, a recent reckoning within the community suggests that in many settings it may not be the most appropriate form of AI support [14, 29, 42, 83, 124]. Substantial empirical evidence has demonstrated that SXAI can lead to overreliance, causing users to accept suboptimal or incorrect AI recommendations [8, 13, 24, 50, 93, 111, 123]. Moreover, it may not cater effectively to the diverse cognitive needs of different individuals [13, 42], and it can impede the development of essential decision-making skills over time [14, 15, 42]. In response to these challenges, alternative human-AI interaction techniques, such as cognitive forcing [13], explanation without decision recommendations [42, 82], evaluative AI [83], contrastive explanations with sensible foils [15] and self-explanation [29], are being developed. Each of these interaction techniques aims to address the pitfalls of SXAI, with the goal of eliciting cognitive engagement to reduce overreliance on AI support [13, 29, 83], or to develop decision-makers’ skills in the long-term [15, 42]. Promising as these new techniques are, each comes with their own trade-offs, making it unlikely that any one of them will be the optimal choice in all situations. Instead, there is growing evidence that the choice of the optimal human-AI interaction technique depends on multiple factors, including context (e.g., AI uncertainty [79, 88] or time pressure [105]), interaction objectives (e.g., decision accuracy or human skill improvement [42]), and individual differences (e.g., cognitive motivation [13, 42], actively open-minded thinking [15]).

Informed by these insights and building on the ethos of adaptive user interfaces, we posit that AI support must be personalized and dynamic, adapting the interaction technique to the individual, context, and interaction objective. For instance, such dynamic assistance may prevent human overreliance on AI by withholding AI assistance in cases when the AI is uncertain, rather than offering SXAI for every decision task. It may also show partial support (e.g., only explanations) instead of providing decision recommendations (“the answer”) to encourage deeper cognitive engagement when the objective includes improving human skills [42] or to individuals who are less likely to engage with AI support.

To enable such personalized and dynamic AI support, we cast the problem of supporting human-AI decision-making as a Markov Decision Process (MDP) and learn policies for dynamically selecting the optimal interaction technique using reinforcement learning (RL). RL is a machine learning approach for learning how to select optimal actions in response to observed states, aiming to maximize cumulative rewards over time. In our setting, the RL policy learns to select the most effective human-AI interaction technique (action) based on contextual factors such as AI’s certainty and an estimate of human knowledge with respect to a task instance (state) while optimizing interaction objectives such as decision accuracy or human learning (reward). We propose RL as a particularly appealing approach for optimizing interaction objectives in AI-assisted decision-making due to its ability to model objectives that are sparse or harder to capture (like human learning) as part of the reward, to capture human-centric (e.g., human’s skill, motivation) and contextual factors (e.g., AI’s uncertainty) as part of the state space, and adapt the support with an action space comprised of different types of human-AI interaction techniques effective for specific contexts. Unlike supervised learning or contextual bandits (a form of RL), which assume dense rewards or one-shot decision-making, full RL is well-suited for modeling human-AI interactions because it captures how actions influence both immediate outcomes and future states, accounting for behavioral adaptations [104] and the cumulative effects of AI support on the decision-maker over time.

In this paper, we employ RL to optimize *decision accuracy* and *human learning* about the task as two important objectives to optimize in AI-assisted decision-making, and as examples of dense (accuracy, which can be measured for every task instance) and sparse (human learning, which can only be measured intermittently) rewards in our proposed approach. We leverage offline RL to derive optimal support policies from existing datasets of human-AI decisions with various AI assistance types, offering a safer alternative to real-time exploration by enabling pre-deployment inspection of the policies and mitigating the significant risks and costs of real-time exploration in actual decision-making scenarios. Drawing on insights from prior work, we construct (i) an action space with four different assistance types that may be effective to optimize accuracy and learning objectives and (ii) a state space that, along with relevant contextual factors (e.g., AI uncertainty), includes human-centric factors and individual differences, such as people’s Need for Cognition (NFC) — a trait that reflects how much an individual enjoys thinking [17] and has been shown in previous studies to significantly predict engagement with AI-provided information [13, 42].

To train the RL policies, we first conducted a data collection study (N=142) in which participants made sequential decisions related to an exercise prescription task. In the data collection study, participants interacted with an exploratory decision-support policy that sampled AI assistance types uniformly. From these interaction data, we applied Q-learning [115] to learn policies that optimized accuracy (immediate accuracy on the task), human learning (accuracy on post-intervention questions answered without AI support), or a combination of both. We tested three broad hypotheses: (1) adaptive policies would outperform or perform as well as static baselines such as SXAI on their target objectives; (2) individuals with different cognitive traits (i.e., Need for Cognition) would benefit from different forms of AI assistance; and (3) policies optimized for a given objective (i.e., accuracy or learning) would yield better outcomes on that objective than policies optimized for a different one. We evaluated these hypotheses through two methods: a computational analysis of the learned policies and two human-subjects studies comparing our RL-optimized policies to multiple baselines, including SXAI.

Our computational analysis and interpretation of the learned policies revealed that optimal policies are different, in meaningful ways and in line with current understanding of the space, for different objectives, contexts, and people with different levels of Need for Cognition (NFC), providing support to our hypotheses. Examining the policies further led to discovering new insights about the AI-assisted decision-making space. Specifically, we discovered that participants low in NFC are unlikely to request AI assistance when that assistance is offered on demand, in contrast to those with high NFC, who were more than twice as eager to seek this optional information.

To understand the downstream impact of adaptive policies on human-AI decision-making outcomes, we further conducted two human-subject studies (N=316 & N=964) — in which people engaged in decision-making while assisted by our RL-powered adaptive decision support or several baselines, including SXAI. Our results demonstrated that accuracy-optimized policies significantly outperformed all other types of AI support: *one-size-fits-all* approaches, such as SXAI only or explanation without decision recommendation only, or policies that either optimized another objective (human learning) or provided support randomly without considering the state of the human-AI dyad, thereby supporting our hypotheses. On the other hand, the learning-optimized policy led to significantly more learning than the accuracy-optimized policy only for the group low in NFC and only in the first experiment, indicating a weaker signal compared to accuracy and partial support of hypotheses.

The fact that we observed clear improvement in decision accuracy (the main metric of success in AI-assisted decision-making) indicates that the general approach of dynamically adapting human-AI interactions using RL is sound and likely to lead to desired outcomes. The fact that although the policies learned the best available actions to improve learning, the behavioral signal was weaker compared to accuracy is likely related to the fact that the design space of interaction techniques (i.e., actions) for stimulating human learning is very sparse (we are aware of only one technique — explanation without recommendation — validated in one study [42]). Thus, we argue that the results provide evidence in support of the MDP formulation and the general method while also demonstrating the need for further development and validation of human-AI interaction techniques that would robustly support human learning. Overall, our findings present strong evidence that current static, *one-size-fits-all* solutions, like SXAI, are insufficient for achieving optimal human-AI outcomes and that AI assistance needs to be dynamic, changing in response to context, individual differences, and the specified objective.

In summary, this paper makes the following contributions:

- We argue that AI support should be dynamic and demonstrate the potential of offline reinforcement learning (RL) for modeling human–AI decision-making, enabling the development of adaptive policies that tailor assistance to individual differences, contexts, and interaction objectives.
- Our instantiation of the proposed approach is consistently successful in improving the key objective in AI-assisted decision-making— joint human-AI accuracy— achieving even human-AI complementarity, and partially successful in improving human learning.
- We further demonstrate the potential of offline RL as a means to discover insights about the AI-assisted decision-making space.
- We contribute new evidence demonstrating the significance of individual differences in cognitive motivation (i.e., Need for Cognition), as a factor to be taken into account when designing AI systems for decision support.
- Our work opens up a novel research challenge of designing novel explanations and human-AI interaction techniques that optimize learning and human skills along with decision accuracy in AI-assisted decision-making.

## 2 BACKGROUND & RELATED WORK

### 2.1 Human-AI Accuracy in AI-Assisted Decision-Making

#### 2.1.1 Towards Calibrated Reliance on AI in AI-Assisted Decision-Making.

AI is becoming increasingly integrated into decision-making processes, with the assumption that it will enhance decision-makers’ abilities by combining their expertise with AI advice to improve decision outcomes. However, mounting evidence shows that decision-makers struggle to incorporate AI recommendations into their decisions, often either over-relying or under-relying on AI, even when explanations are provided [5, 8, 13, 24, 45, 78, 82, 90, 93, 100, 100, 119].

Recognizing this challenge, substantial efforts have been made to characterize the types of explanations or indicators of uncertainty [24, 54, 77, 96, 111, 119, 122], situations (e.g., the cost-benefit of engaging with AI [111], time pressure [20, 105]) and other settings [47, 65, 95] in which people resort to over- or under-relying on AI and devise interventions that promote calibrated reliance and effective utilization of AI support. These research endeavors can be broadly categorized into pre-task and in-the-moment interventions. Pre-task interventions often involve training or onboarding sessions designed to help individuals construct a mental model of AI [56, 85, 86, 94], develop a self-mental model related to the task [49], or increase human agency by granting them control over input feature selection and algorithmic assistance [26, 64]. In-the-moment interventions, on the other hand, consist of interventions such as explanation [118], interaction [13], meta-information [16, 85], and paradigms [83] that promote effective AI support use during the decision-making process. Some of these interventions can be broadly grouped into evaluation-soliciting decision support, such as Miller’s proposed Evaluative AI paradigm, which presents evidence both for and against a decision *after* the human makes an initial decision [83]. Other approaches involve presenting explanations in the form of questions rather than statements [29], or decision support that incorporates evidence from the literature and presents it alongside AI advice [118]. Related to our work, a nascent branch of in-the-moment interventions includes adaptive strategies that learn to present decision-makers with AI support only when it is deemed beneficial. To identify such instances, these adaptive interventions leverage a model of human decision-makers [79, 88] or learn decision policies with contextual bandits [10]. These strategies, which optimize whether or not to provide support for optimizing immediate accuracy, seem promising for effective AI-assisted decision-making: both Noti and Chen [88] and Ma et al. [79] report human-AI complementary team performance.

#### 2.1.2 The Impact of Different AI Assistance on (Over)Reliance and Cognitive Engagement.

Studies consistently show that simple explainable AI (SXAI), in which people are provided with AI recommendations and explanations, induces overreliance on AI [8, 13, 45]. Previous research in AI-assisted decision-making has put forth that this overreliance on AI stems from superficial engagement with the information provided [12, 13, 42]. People overrely on AI recommendations as they fail to cognitively engage with the presented AI suggestion and explanation. Research from learning sciences has long established that cognitive engagement with information is essential for learning [99]. As such, AI assistance types that induce overreliance will potentially hurt cognitive engagement and subsequently learning. Whereas AI assistance types that induce cognitive engagement will help people critically evaluate information and disregard incorrect AI suggestions, resulting in both increased learning and reduced overreliance.

One assistance type that previous work suggests enhances cognitive engagement is providing people with AI explanation only [42]. The underlying hypothesis is that providing people with AI explanations only, as opposed to showing them AI recommendations and explanations, invokes more cognitive engagement because people have to make the cognitive jump of getting to a final decision from the given information rather than being “served the answer”. Another form of AI support that previous work has shown to reduce overreliance, and possibly induce cognitive engagement, is letting people choose whether or when they want to see AI recommendations and explanations (i.e., on demand) [13]. By tapping into people’s curiosity for viewing the AI advice and allowing them control over when or whether to view the AI suggestion, such assistance may elicit cognitive engagement with the AI-provided content. We included these two designs in our study, as assistance types that had the potential to support cognitive engagement, and thus, human learning about the domain.

### 2.2 Human Competence, AI, and the Future of Work

The anticipated large-scale deployment of AI-powered decision aids is likely to transform many jobs. There are moral and economic reasons to look for ways to deploy these technologies in a manner that complements workers and enhances their abilities, rather than diminish their roles or replace them without offering new opportunities [1, 102]. However, many of the current deployments of AI-powered decision support systems are likely to negatively alter the existing workplace dynamics. For example, when workers rely on one another for help with difficult decisions, such help typically results in *incidental learning* that enables workers to develop their skill over time [9]. In fact, some researchers argue that a large fraction of learning that occurs in organizations happens via informal channels such as incidental learning [80, 81] and such learning is essential not just for workforce development but also for worker well-being [30]. However, receiving help from systems that offer a decision recommendation accompanied by an explanation does not seem to result in incidental learning [42]. Instead, it can lead workers to incorrectly increase their confidence in their ability to perform similar tasks in the future [35, 36].

As AI increasingly assists knowledge workers in decision-making by providing decision recommendations, a critical question arises: how will such assistance affect decision-makers’ work motivation in the long term? Self-determination theory (SDT) — a macro theory for understanding human motivation — may provide insights into how AI support systems may affect decision-makers’ long-term motivation in the workplace [30]. Originating from studies on intrinsic and extrinsic motivations and subsequently broadening its scope to encompass investigations in areas such as work organizations and various aspects of daily life, SDT identified *competence* as one of the three psychological needs which mediated workers’ performance and well-being in the workplace. Competence reflects a person’s drive to be effective and skilled in their work environment. It involves exploring and engaging with surroundings and taking on challenging tasks to assess and improve abilities. Meanwhile, the current design of AI support with recommendation and explanation (SXAI) may be inadvertently undermining decision-makers’ competence. While the longitudinal impact that SXAI has on competence is yet to be studied, evidence from automation [28] and a recent study in AI-assisted decision-making [42] suggest that decision recommendations may hinder decision-makers’ learning and skill improvement. Given the critical standing of competence in workers’ motivation, well-being, and performance, we posit that supporting workers’ skill improvement and knowledge acquisition (along with their decision accuracy) is a critical human-centric objective for the design of AI for decision support.

### 2.3 Adaptive Interfaces

Our work builds on a substantial body of research on adaptive interfaces & interventions: systems that dynamically adjust layout, content, or interaction techniques to better suit different users, contexts, or devices. Researchers have pursued adaptation for a range of goals, including increasing user engagement [23, 52], supporting learning [33, 121], and improving interaction efficiency [39, 40, 108]. For instance, recommender systems adapt content (e.g., videos) to sustain engagement [52]; adaptive tutoring systems modify scaffolding to promote learning gains [33]; and HCI research has focused on adapting interface layout (i.e., the spatial arrangement of elements on the screen) [40], interaction techniques (e.g., to support different devices and input modalities such as mouse and touch) [61], and visual aesthetics [38] to improve interaction efficiency, accessibility, and user satisfaction.

Achieving these goals has required a corresponding evolution in how adaptive interfaces are represented and generated. Computational models and automatic interface generation have long been central concerns in HCI, with researchers developing increasingly flexible and scalable techniques to move beyond rigid, hand-coded designs. Early work explored specifying interfaces through constraints [11], declarative modeling languages [106], and interactive tools for direct manipulation of graphical elements [120]. Building on these foundations, later work framed interface design as an optimization problem [40, 43, 44], enabling fully autonomous, run-time generation of interfaces tailored to users whose devices, tasks, preferences, or abilities were not well supported by static designs. More recently, advances in deep learning have enabled data-driven approaches to interface representation and generation [69, 114, 117], further expanding the possibilities for real-time, personalized adaptation. Bayesian optimization has also been applied for human-in-the-loop adaptation, including visual design optimization [62], exploration of interaction design spaces [73], and maximizing engagement through adaptive game difficulty [58].

Related to our work, reinforcement learning (RL) has emerged as a powerful computational approach for interface adaptation, particularly in pursuit of interaction efficiency. For example, Todi et al. [108] use RL to dynamically reorder menu items based on simulated user interactions; Langerak et al. [66] introduce a multi-agent RL framework where one agent simulates user behavior (e.g., clicking, navigating menus), and another adapts the interface to surface the most relevant items; and Lingler et al. [76] use RL to model user attention in dual-task environments, enabling adaptive interventions that reduce cost of task switching. In line with computational rationality as a theory of interaction [89], across this body of work, RL is primarily used to model user behavior either to better understand it or to enable low-level interface interventions for improving interaction efficiency.

RL has also been applied to support adaptive tutoring in educational contexts, where the goal is to improve learning rather than interaction efficiency. Here, instructional interactions are typically modeled as Markov Decision Processes (MDPs): a student’s cognitive state defines the state space, instructional activities (e.g., flashcards, videos, problem steps) serve as actions, and the reward function combines the cost of actions (e.g., time) with educational outcomes such as skill acquisition [7, 33]. Another related thread of work in mobile health (mHealth) uses RL for behavior change through just-in-time adaptive interventions (JITAIs). While these systems operate in a different domain, they share with our work a core emphasis on influencing human cognition and behavior through context-aware, personalized interventions. They aim to promote healthy behaviors — such as increasing physical activity [71], encouraging dental hygiene [109], or reducing substance use [46] — by delivering personalized prompts at opportune moments. The state space typically includes contextual data inferred by mobile use (e.g., time, location, recent behavior), and actions are often motivational messages or behavioral nudges designed with domain experts.

Our work extends this body of research in five key ways. First, we apply RL in the context of AI-assisted decision-making, where an additional agent — the AI — introduces uncertainty and influences human decision processes and cognitive engagement, requiring new forms of interface adaptation. Unlike traditional adaptive interfaces, which adjust deterministic elements like layout or navigation structure, interaction with AI involves stochastic outputs that can be ambiguous, fallible, or misleading. This makes the adaptation problem fundamentally different: the system must account not only for user behavior, but also for the probabilistic nature of the AI’s suggestions and their impact on human reliance, understanding, and decision quality. Second, instead of adapting layout or interface elements, we focus on adapting the human–AI interaction technique, that is, the design and structure through which information is exchanged between the user and the AI. Third, our objective shifts from optimizing low-level interaction metrics (e.g., click speed or search time) to enhancing semantic accuracy and supporting incidental user learning during complex decision tasks. Fourth, unlike traditional educational settings where the user’s explicit goal is to learn, our setting involves learning as a *byproduct* of interaction with AI, requiring different adaptation strategies. Finally, from a technical standpoint, we employ a model-free, offline RL approach that learns policies directly from interaction data, without explicitly modeling the underlying MDP — unlike much prior work on adaptive interfaces, which often relies on model-based RL.

### 2.4 Offline Reinforcement Learning

Reinforcement learning (RL) is a popular approach to designing intelligent systems that learns by interacting with an environment, and can be divided into two categories based on the data collection strategy: online and offline RL [103]. Online RL entails learning optimal policies through direct interaction with the environment, either in the real world or within a simulated setting, in real-time. Whereas, offline RL involves learning optimal policies from a previously-collected interaction dataset. One of the main advantages of online RL is its ability to adapt to changes in the environment and to learn in real time, making it well-suited for applications that require continuous adaptation. Online RL has been employed in various applications of user interfaces (e.g., menu selection [70], visual search [25], typing [53]) in which policies are typically learned by interacting with a computational model or simulation of the user behavior. Online RL with real users (i.e., in a real-world environment) can be risky (due to exploratory actions taken in real time), computationally expensive (especially in time-constrained settings), and data-intensive, with data collection in the real world often being costly. Offline RL, on the other hand, is safer and less computationally expensive, as it learns from a fixed dataset before the policy is deployed. However, offline RL may not generalize well to new environments (such as when parts of the environment are not sufficiently explored in the offline training dataset) [68]. We opted learning policies from actual human-AI interaction data as opposed to simulations. With faithful computational models or simulators of human-AI interaction, learning policies with simulated data would also be possible. However, our understanding of human-AI decision-making as a field is still in its infancy, therefore, any assumptions baked into a computational model may likely turn to be incorrect, and thus, yield flawed policies (e.g., One such assumption was that explanations would help people calibrate their reliance on AI; they generally do not [8].). Because of the real-world constraints and cost of employing online RL for crowdsourcing studies with actual users, we chose an offline RL setup and collected data accordingly. We learned our policies using Q-Learning on data that was previously collected using an exploratory policy.

Q-Learning [115] is a prominent off-policy algorithm in RL for data collected in an offline setting. It estimates the expected long-term reward of each state-action pair (the “Q-value”) in the RL environment. Q-learning estimates a state-action pair’s Q-value by iteratively updating it based on the observed reward and the estimated value of the next state. Once Q-learning converges, the optimal policy is the action with the highest value for each state. One of the key advantages of off-policy algorithms is that they can learn from data collected using any policy, which allows the agent to learn from potentially suboptimal but diverse behaviors. For example, collecting data using an exploratory policy can help to prevent the agent from getting stuck in suboptimal behavior and to learn from potentially rare but valuable experiences.

### 2.5 Need for Cognition

Need for Cognition (NFC) is a stable personality trait that captures how likely a person is willing to engage in non-required cognitively demanding activities [18]. In other words, it reflects a person’s general cognitive motivation. Across, numerous fields such as skill acquisition, processing of information in advertising and in health communication, or web usage, there is consistent evidence that high need for cognition is associated with seeking out more information and processing that information more deeply [22, 74, 101, 110, 112, 116]. In HCI literature, there is also initial evidence that people high in NFC are more likely than those with low NFC to exert cognitive effort when interacting with complex digital systems and to benefit more from the more complex features [21, 41, 110]. In the area of AI-supported decision-making, previous work found that compared to individuals with low NFC, those with high NFC make better decisions [13] and benefit more from novel human-AI interaction techniques such as cognitive forcing [13] or receiving only explanations without decision recommendations [41]. For those reasons, we identified need for cognition as a particularly relevant dimension of individual differences.

## 3 OVERVIEW: APPROACH & HYPOTHESES

In this work, we sought to build computational models that dynamically select interactions based on (1) the desired objectives of AI-assisted decision-making, (2) the individual differences among the decision-makers, and (3) the relevant contextual factors. For this paper, the two objectives we chose to optimize for were people’s immediate decision accuracy and their longer-term learning about the task (as measured by their accuracy on distal tasks where they receive no AI support). The individual difference as a relevant human-centric factor for which we personalized was people’s Need For Cognition (low vs. high), drawing on results from prior work that have shown it to be an important predictor of engagement with different forms of AI support [13, 42]. Informed by previous work discussed above, the contextual factors we considered were the type of decision-making instance, AI’s uncertainty, decision-maker’s competence, and knowledge related to the concept in question.

Our setting was a sequential decision-making task, in which the same individual made a large number of decisions in the presence of an AI-powered decision support system. On any particular decision task instance, the decision support system could support the human decision-maker using one of several methods (no support, showing a decision recommendation with an explanation, showing just an explanation but offering no decision recommendation, or allowing the person to request support on demand), each with a different possible impact on a person’s decision performance in the moment and on their learning about the task domain. We formulated the problem of personalizing AI support as a Markov Decision Process, employing reinforcement learning (RL) algorithms to learn optimal policies that select sequences of interactions by accounting for the context and individual differences when optimizing the desired objectives (See Section 4.2).

Our work was guided by the following three broader hypotheses about adaptive support in AI-assisted decision-making which we further broke down into specific hypotheses in subsequent sections:

1. Optimized policies will result in human performance on the target objectives (i.e., accuracy and learning) that is as good or better than the performance achieved with baseline policies that do not consider contextual factors.
2. People with different levels of Need for Cognition (NFC) will benefit from different types of AI assistance for objectives that require cognitive engagement (i.e., learning).
3. For each group of people based on their level of NFC, policies optimized for a target objective (i.e., accuracy or learning) will result in better human performance on that objective than policies optimized for another objective.

The remainder of the paper is structured as follows. In Section 4.2, we introduce the decision-making task and present our Markov Decision Process (MDP) formulation of human-AI decision-making. Section 5 details our approach to learning policies, including data collection with an exploratory policy, training, and computational evaluation. Sections 6 and 7 present two human-subject studies that evaluate the downstream effects of the adaptive policies on human-AI decision-making. Finally, Section 8 synthesizes findings from both the computational evaluation and the user studies.

## 4 TASK & PROBLEM FORMULATION

### 4.1 Exercise Prescription Decision-Making Task

**Fig. 2.**
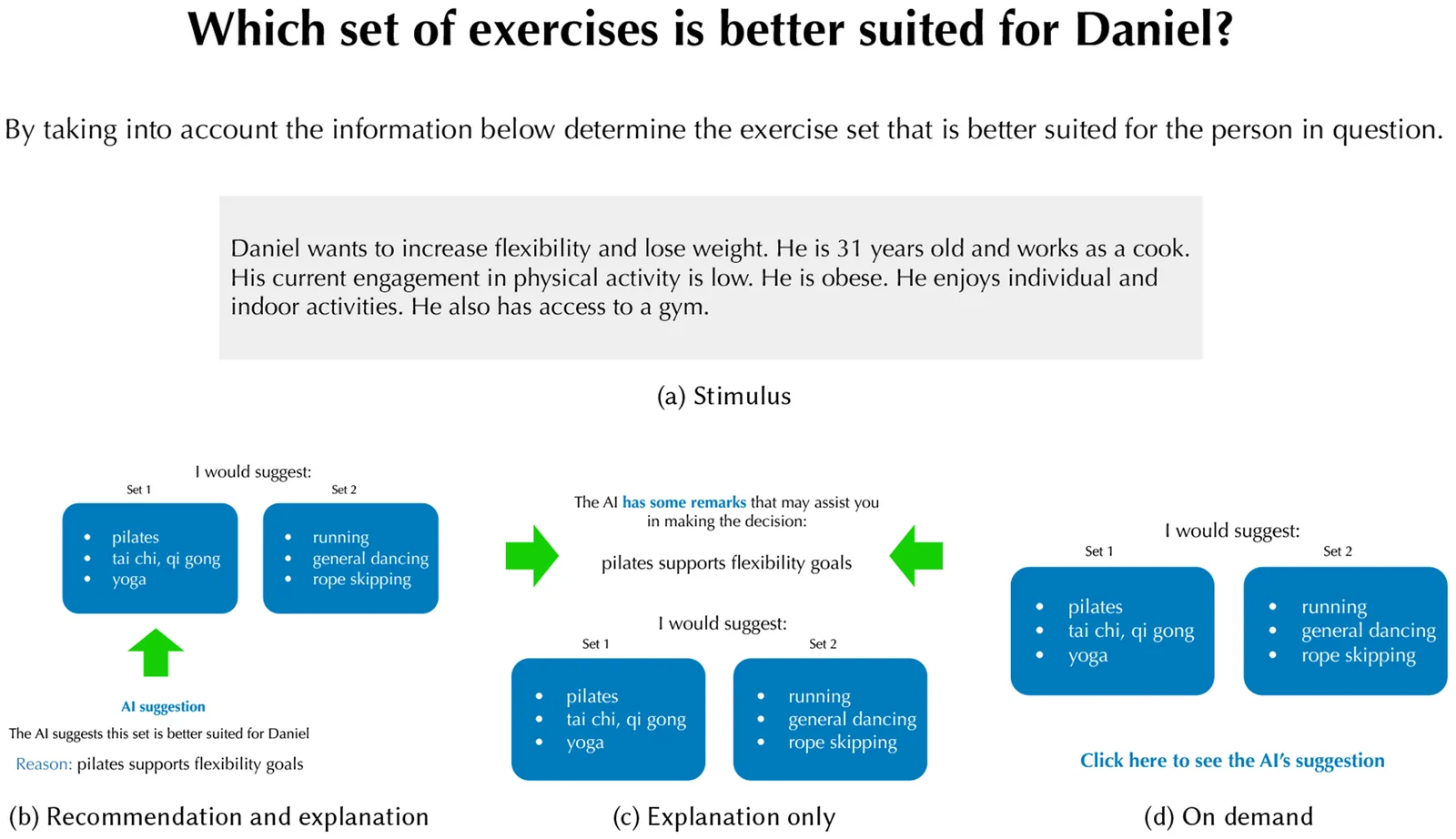
An example of the exercise prescription decision-making task with different types of AI assistance (i.e., actions). Participants were assisted in choosing between the two sets of exercises as depicted for different conditions. In the *No-AI* condition (not shown) participants were not provided with any AI assistance.

Figure 2 depicts an example of the decision-making task. We aimed to create a decision-making task that would be accessible to laypeople on crowd-sourcing platforms, while also inducing similar cognitive challenges as high-stakes decision-making tasks such as those encountered in clinical decision-making. To accomplish this, we teamed up with a kinesiology expert (also a co-author of this paper) and created decision instances for an exercise recommendation task. The task involves selecting the most suitable of two exercise sets for a (fictional) person based on their description, goals, and preferences. Exercise recommendation as a decision-making task is accessible to a broad audience, yet poses similar challenges to treatment selection in clinical settings. When choosing a treatment, clinicians weigh various factors such as the patient’s condition, treatment preferences, side-effect tolerance, and constraints. Similarly, in exercise prescription, each exercise type may interact differently with the person’s goals, health factors, preferences, capabilities, or resources.

We generated 44 vignettes of fictitious people by randomly sampling their demographics (age, gender, BMI, physical activity level, occupation, smoking status) and accordingly manipulating the following six factors which were deemed important for exercise prescription by the expert: (1) their maximal or target intensity (based on demographics), (2) their exercise goal (*e.g.*, building muscles, weight loss, flexibility), (3) their exercise preference (*e.g.*, indoor/outdoor, group/individual), (4) their resource availability (*e.g.*, access to a swimming pool), (5) their medical condition if any (based on their age and gender), and (6) their susceptibility to experience adverse events during the exercise (based on their age and medical condition).

To build an exercise repository from which to recommend exercises to the fictitious people, we curated a list of 60 leisure time exercises from a comprehensive compendium which consisted of physical activities ranging from different exercises (*e.g.*, sports) to everyday activities (*e.g.*, housework, occupational activities) [3]. Given a fictitious person and the list of exercises, the expert selected a list of *optimal* and a *suboptimal* exercises for the person. The *optimal* and *suboptimal* choices differ substantially in at least one of the four concepts (intensity, goal, medical condition, and safety), which rendered the optimal exercise set choice superior to the suboptimal choice. For example, for a fictitious person whose goal is to increase flexibility, optimal exercises such as *pilates* and *yoga* would account for their goal, while suboptimal choices such as *running* or *rope skipping* may fit their other needs but would not support their goal.

#### Generating explanations.

In order to generate effective explanations, we carefully crafted them to highlight the specific concept of one of the exercises that resulted in one exercise set being more optimal than the other. Thus, if one set of exercises was superior to the other due to the medical condition, the explanation would highlight the feature of one of the exercises in the superior set that makes it suitable for the person in question. For example, if the person in question had osteoporosis and the superior set included low-impact exercises such as *swimming*, the explanation would take the form: *swimming is suited for people with osteoporosis because it is low-impact*.

#### Generating incorrect AI recommendations.

Our experiment aimed to recreate realistic scenarios in which an AI model assisting individuals during decision-making might not always be accurate. To simulate such situations, we used a subset of the questions (25% for the data collection study and 28.6% for the evaluation study) in which the AI assistance presented the suboptimal choice as the recommendation and generated a factually correct but unhelpful explanation. The explanation highlighted an arbitrary concept for which the suboptimal exercise was superior to the optimal exercise. This approach allowed us to test participants’ ability to recognize the suboptimal concept and make a sound decision despite the explanation provided. For example, for the same fictitious person as before whose goal was to increase flexibility, optimal exercises such as *pilates* and *yoga* would account for their goal, while suboptimal choices such as *running* or *rope skipping* may fit their other needs but would not support their goal. An unhelpful explanation of the incorrect AI suggestion would be: *running maximizes the intensity the person is capable of exerting*.

### 4.2 Problem Formulation

Our goal was to optimize for accuracy on the current task instance as a proximal outcome (dense reward), and human learning about the task domain as a distal outcome (sparse reward). To this end, we formulated the problem of selecting the appropriate decision support method for a given task instance as a reinforcement learning (RL) problem. RL is particularly well suited to this setting because it models how actions influence not only immediate outcomes but also future states — capturing behavioral adaptations and the cumulative effects of AI support that unfold over time [104]. In contrast, supervised learning assumes static input-output mappings under full supervision, and contextual bandits treat each decision as independent. These approaches cannot account for sparse rewards and how different forms of assistance might shape future engagement, performance, or receptivity. This makes RL uniquely capable of modeling the dynamic, temporal nature of human-AI interaction.

Let the Markov Decision Process (MDP) be defined by the tuple (𝒮,𝒜,𝒯,ℛ,𝛾) where 𝒮 is the state space, 𝒜 the action space, 𝒯:𝒮×𝒜→Δ(𝒮) is the probability transition function, ℛ is the reward, and 𝛾 corresponds to the discount factor. A policy π:𝒮×𝒜→[0,1] assigns each state s∈𝒮 a distribution over actions π(a|s), where a∈𝒜. In our setting, 𝒮,𝒜,ℛ are designed as follows:

- **State.** In order to capture the current state of the human-AI decision-making dyad, we consider the concept under investigation, AI’s accuracy, and the decision-maker’s level of knowledge and their propensity to engage in analytical thinking. Specifically, at each time step *t*, we represent the state as *s_t_* = [*nf c_t_, c_t_, u_t_, h_t_, k_t_*], where:
  - *nf c_t_* denotes the decision-maker’s Need for Cognition (NFC) [17], a personality variable that measures intrinsic motivation to think. To capture this, we asked participants to answer four questions with the highest factor loading from the NFC questionnaire [17], and then categorized their score as *low* if it fell below the median or *high* otherwise.
  - *c_t_* represents the concept that is being queried at time step *t*, which makes the optimal exercise superior to the suboptimal exercise for the given vignette. For example, if the task is to choose between walking and ice-skating, and the relevant factor that tips the balance in favor of walking is safety, then safety is the concept that is being probed. In total, four different concepts may be queried: intensity, goal, safety, and condition.
  - *u_t_* represents the model uncertainty regarding its prediction for the current question, which can be modeled as a continuous variable. However, we utilize a binary variable based on the ground truth of the AI’s accuracy (i.e., correct or incorrect). We acknowledge that this simplification is not realistic; nevertheless, it minimizes potential confounding variables and enhances our confidence in the overall findings.
  - *h_t_* captures the decision-maker’s average knowledge of concept *c_t_* up to time step *t* – 1. It is inferred in real-time as the decision-maker’s average accuracy over all previous questions about concept *c_t_*, discretized to a binary variable with a threshold of 0.6.
  - *k_t_* captures the decision-maker’s knowledge about the task, measured by their performance on three initial questions with no assistance at the beginning of the study. We classify their knowledge as either *low* or *high*, depending on whether their average performance was below or above 0.5.
  With four possible values for the concept and two values for each of the other four dimensions, our state space consisted of a total of 64 possible states.
- **Action space** is comprised of four different interface presentations.
  - No assistance: participants receive no AI assistance.
  - Explanation only: the AI explanation is shown with no recommendation. Previous work suggests that showing explanations only fosters learning about the domain [42].
  - AI explanation and recommendation (SXAI): both AI recommendation and explanation about the decision are shown. Numerous studies have demonstrated such a design to increase accuracy because of (over)reliance on the AI [8, 13, 42]. Note that throughout the paper for clarity, we refer to the policy that presents AI recommendation and explanation on each decision as simple explainable AI (SXAI), whereas to the type of assistance as *recommendation and explanation*.
  - On demand: the AI recommendation and explanation are shown upon request. As this action elicits curiosity about the AI’s prediction, we hypothesized that it would increase cognitive engagement with the task [13], thus learning as well.
- **Reward** is multi-objective, seeking to maximize a combination of accuracy (whether the answer in the current question is correct) and learning (whether the provided answer in the later test question with no AI assistance is correct). An answer is considered correct when it matches the expert ground truth. We denote the reward as follows:
(1) $$r_{t}=(1-\lambda )p_{t}+\lambda d_{t}$$
  where *p* indicates the *accuracy* outcome, *d* indicates the *learning* outcome, and *λ* is the weighing hyperparameter over these two outcomes. For learning, we receive the reward only at test time. However, in offline RL all the data is accessible, allowing assignment of credit to an action at the current time for its impact on learning that is assessed in the future. In other words, if a concept was presented with an action *k* at time *t_i_*, we assign to the action *k* the future learning reward that is measured at test time ^1^ *t_j_*, where *j* > *i*.

The data of each participant *i* is considered as an episode:

$${𝒟}_{\rangle }=\{s_{1},a_{1},s_{2},a_{2},\ldots ,s_{t},a_{t},\ldots ,s_{T+1}\}$$

where *T* is constant and presents the length of the experiment (24 questions), $S_{t}\in 𝒮$, $A_{t}\in 𝒜$, and $R_{t}\in ℛ$ (the reward received at time step *t*). Note that the underlying transition probabilities $T\left(s^{\prime }|s,a\right)$ are not directly known in model-free offline policy learning. Instead, the agent infers the dynamics of the environment from the observed transitions in the dataset [68].

We operate under the Markov property assumption (“the future is independent of the past given the present”).

## 5 METHOD

Having formulated the problem as a reinforcement learning (RL) problem, we sought to collect data from which to learn policies that optimize for the decision-makers’ proximal and distal benefits when assisted by an AI.

### 5.1 Data Collection with an Exploratory Policy

The purpose of the data collection study was to collect human-AI decision-making data to inform the development of interaction policies aimed at optimizing either immediate decision accuracy or the learning of decision-makers.

#### 5.1.1 Task.

Participants completed a series of 33 questions, in which each question concerned a vignette about a fictitious character; participants were tasked with selecting which of two sets of exercises was optimal for the character in question.

#### 5.1.2 Experiment design.

We sought to design an experiment that would enable modeling the impact of various types of assistance on participants’ immediate accuracy and their learning about the domain. As such, each participant was randomly assigned to a subset of three different concepts from a pool of four concepts and answered 11 questions per concept, a total of 33 questions. This design choice was informed by work in educational research that highlights the importance of repeated exposure to a concept for effective learning [60].

**Fig. 3.**
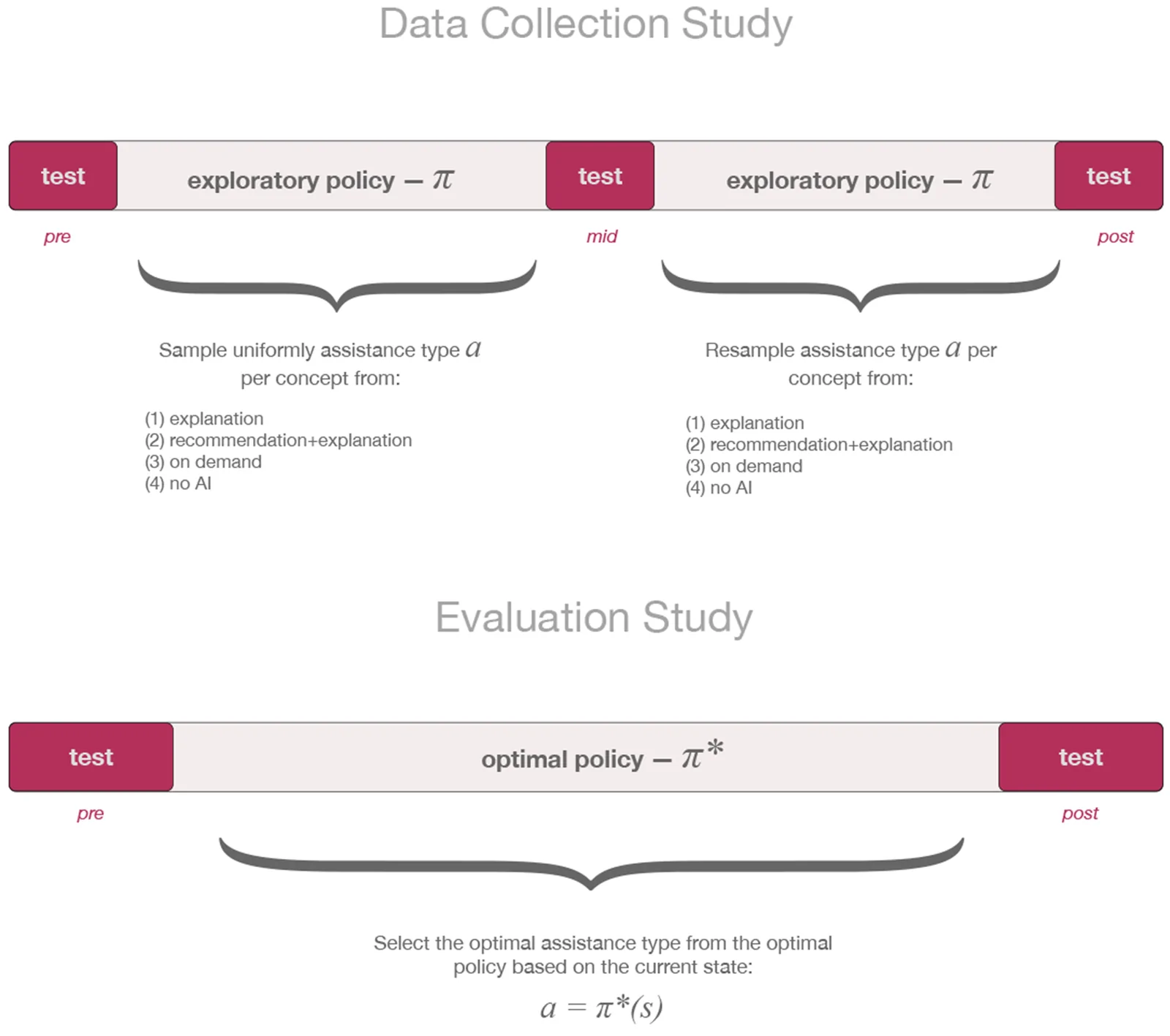
An overview of the experiment flow for the data collection and evaluation studies. In the evaluation studies, participants were randomly assigned to one of the optimal policies (that matched their NFC level) or a baseline policy.

Figure 3 illustrates the experiment design, which consisted of three test blocks (*pre, mid*, and *post*) and two intervention blocks (*first* and *second*). Participants received no AI support on test blocks, which served as evaluation points to measure their initial knowledge and subsequent learning about the domain. On a given intervention block, a concept was presented only with one type of assistance (randomly picked), to isolate the effect of the assistance type on learning. The intervention blocks were preceded and followed by a test block. Notably the *mid* test block served as a *post* intervention evaluation for the first intervention block and a *pre* intervention evaluation for the second intervention block. Each test block consisted of one question per concept, a total of 3 questions. Each intervention block consisted of 4 questions per concept, a total of 12 questions.

The AI system had an overall accuracy of 75% (three out of 12 intervention questions per block were incorrect). The order of the questions was randomized for each participant and the questions in which the AI made incorrect suggestions were picked randomly. We did, however, ensure that AI was uniformly wrong across concepts, i.e., each of the three incorrect questions per block belonged to a different concept.

In an intervention block, each of the three concepts was quasi-randomly matched with an AI assistance type from the four available AI assistance types – *no AI support, on demand, explanation, recommendation+explanation*. We refer to the procedure as *quasi* random because we purposefully sampled *no AI* less often than the other forms of AI support. Throughout a given block, a concept was presented only with the assistance type it was assigned to. The same process of assigning concepts to AI assistance types was repeated for the second intervention block. This design choice enabled collecting a larger amount of data per participant to model the impact of different assistance types on immediate accuracy and learning.

#### 5.1.3 Participants.

Given that there are no guidelines for determining the sample size from which RL-based algorithms would reliably capture a signal from the data, the sample size was informed by MRT-SS Calculator, a sample size calculator for micro-randomized trials [72]. A sample of 139 participants is required to attain 80% power with a significance level of 0.05, and 24 (intervention) decision points (i.e., questions). ^2^ We recruited a total of 150 participants for the data collection study via Prolific, an online recruitment platform. We retained 142 participants for analysis, contingent on their performance surpassing the attention check threshold. Given that the task involved making decisions based on the comprehension of vignettes, participation was limited to adults in the United States who were fluent in English (for detailed demographics, see Appendix Table 2). Each participant was compensated 2.4 USD (12.72 USD per hour).

#### 5.1.4 Procedure.

Our online study was administered through Prolific. Participants were initially provided with a brief overview of the study, and if they agreed to participate, they were directed to an informed consent form. Participants were then required to complete a Need for Cognition (NFC) questionnaire, which included the four items with the highest factor loading from the widely used 18-item instrument [17], as identified in previous work [41]. Additionally, participants were given the option to complete a demographics form.

Following the completion of these forms, participants were presented with detailed instructions about the task. They were also informed that at times they may receive assistance from an AI that was still under development and was prone to error. The task consisted of answering 33 questions involving exercise recommendation for a series of fictitious characters. At the study’s conclusion, participants were asked to report any technical issues they encountered, as well as any instances of cheating. Lastly, participants were provided with feedback on their performance during the study.

#### 5.1.5 Approvals.

All experiments reported in this paper were approved by our institution’s Internal Review Board, under the protocol number [anonymized for review].

### 5.2 Learning the Optimal Policies

From the collected data, our goal was to learn optimal policies with which to provide assistance to the human decision-maker in order to optimize their accuracy or learning.

We opted for offline reinforcement learning approaches, which aim to learn the optimal policy π* from an exploratory, behavioral policy *π_β_*. In our setting *π_β_* is the quasi-uniform policy with which data was collected. We picked Q-learning, as a model-free off-policy RL algorithm that does not require state transition probabilities.

The Q-function was learned by iterating,

(2) $$Q\left(s,a\right)\leftarrow Q\left(s,a\right)+\alpha \left(r+𝛾\;\underset{a^{\prime }}{\max}\;Q\left(s^{\prime },a^{\prime }\right)-Q\left(s,a\right)\right)$$

with the next state in the episode *s′* (i.e., next question for a participant) and learning rate *α*. We used learning-rate decay on *α* to speed up convergence, set at a value of 0.1/*i*, where *i* was the number of iterations passed. In our setting, the reward *r* is substituted by (1 – *λ*)*p* + *λd* as in equation 1.

#### Optimizing accuracy.

When optimizing for accuracy, we myopically seek to maximize the immediate accuracy, disregarding both learning and future rewards. As such, we set both the hyperparameter *λ* = 0 and the discount factor *𝛾* = 0. In this way, the algorithm learns to select actions by solely considering the immediate decision accuracy.

#### Optimizing learning.

On the other hand, optimizing learning requires consideration of both the learning (*λ* = 1) and the future rewards (*𝛾* = .99).

For each objective, we ran 200 iterations over the 142 episodes (i.e., participants), achieving convergence of the *Q*-table. Finally, we constructed the optimal policy by picking the optimal action greedily, $\pi^{*}(s)=\underset{a}{\arg\max}Q\left(s,a\right)$ for each state *s*, thereby forming a mapping of states to corresponding optimal actions.

### 5.3 Computational Evaluation of the Learned Policies

First, we examined computationally the learned policies through the lens of the broader hypotheses regarding differences among different objectives and different groups of NFC. Sections 6 & 7 describe the subsequent evaluation of the policies via two user studies.

#### 5.3.1 Hypotheses & Research Questions.

With the computational evaluation, we sought to answer the following hypotheses and research questions:

- **H1a:** Optimized RL policies will differ from fixed policies (e.g., simple explainable AI policy). Specifically, at least one policy for each NFC group will employ a set of interactions that differs significantly from a fixed policy that always uses simple AI recommendation and explanation interactions.
- **H2:** RL policies optimized for improving human learning, as an objective which requires cognitive engagement, will employ different interactions for individuals low in NFC than RL policies optimized for improving the learning of individuals high in NFC.
- **RQ1:** Will RL policies optimized for improving the immediate decision accuracy of individuals low in NFC employ different interactions than RL policies optimized for improving the immediate decision accuracy of individuals high in NFC?
- **RQ2:** Will RL policies optimized for improving the learning of each group of NFC employ different interactions than RL policies optimized for improving immediate decision accuracy?

**Fig. 4.**
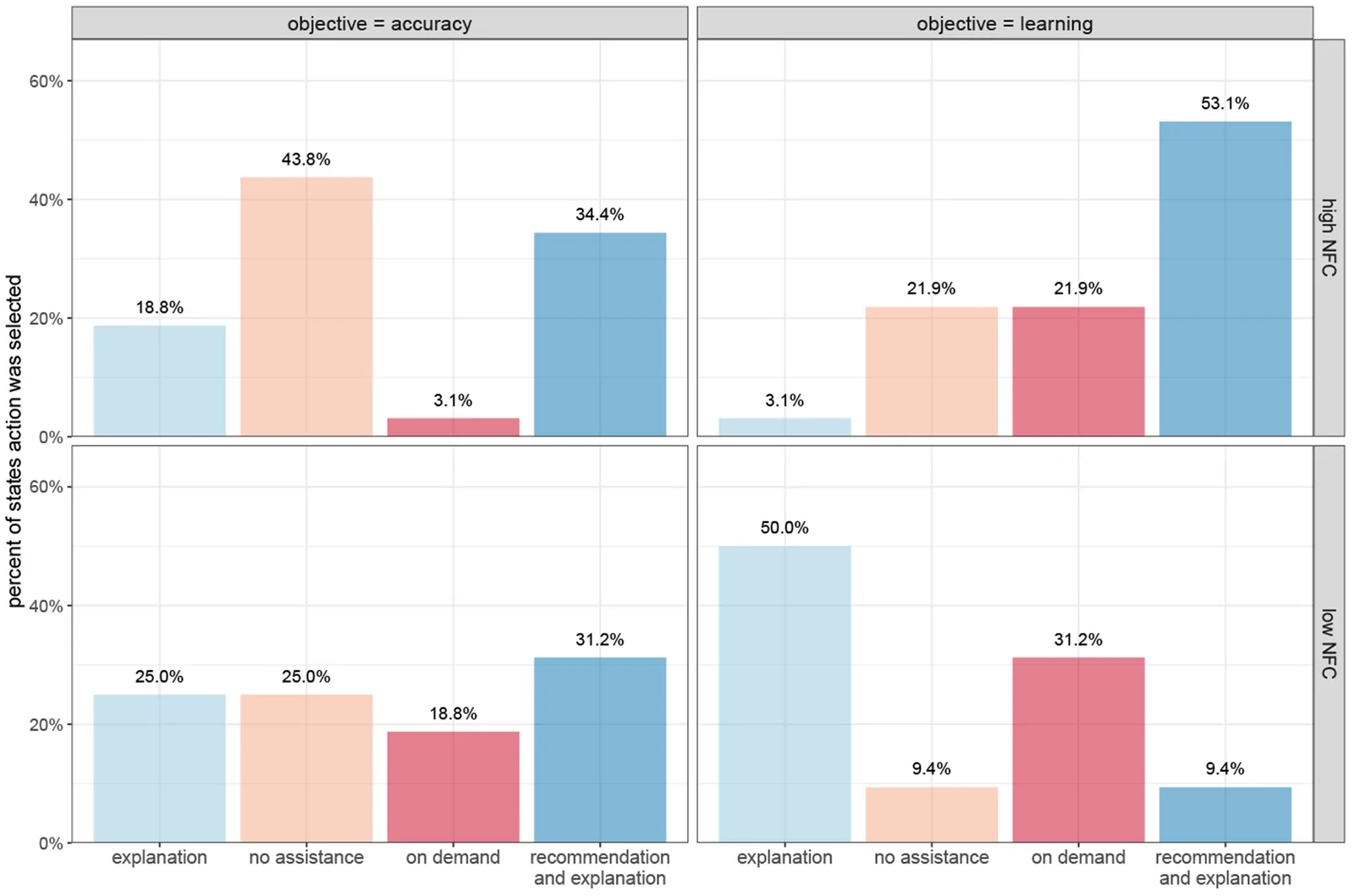
Distributions of types of AI assistance selected by the optimal policies for different objectives and NFC groups. Each bar in the figure represents the percentage of states in which an action was the top action, with the numerator being the number of states where the action was the top choice and the denominator being the total number of states in the analysis.

Figure 4 depicts the distributions of the types of AI assistance for different objectives and different NFC groups.

#### 5.3.2 Results.

In line with **H1a**, both the *accuracy* and the *learning* policies differ substantially from any fixed policy, such as the SXAI policy, which employs only the recommendation and explanation action. We are unable to conduct a *χ*^2^ test due to its inapplicability for distributions containing zero occurrences, which is the case for all actions other than *recommendation and explanation* in the SXAI policy.

**Fig. 5.**
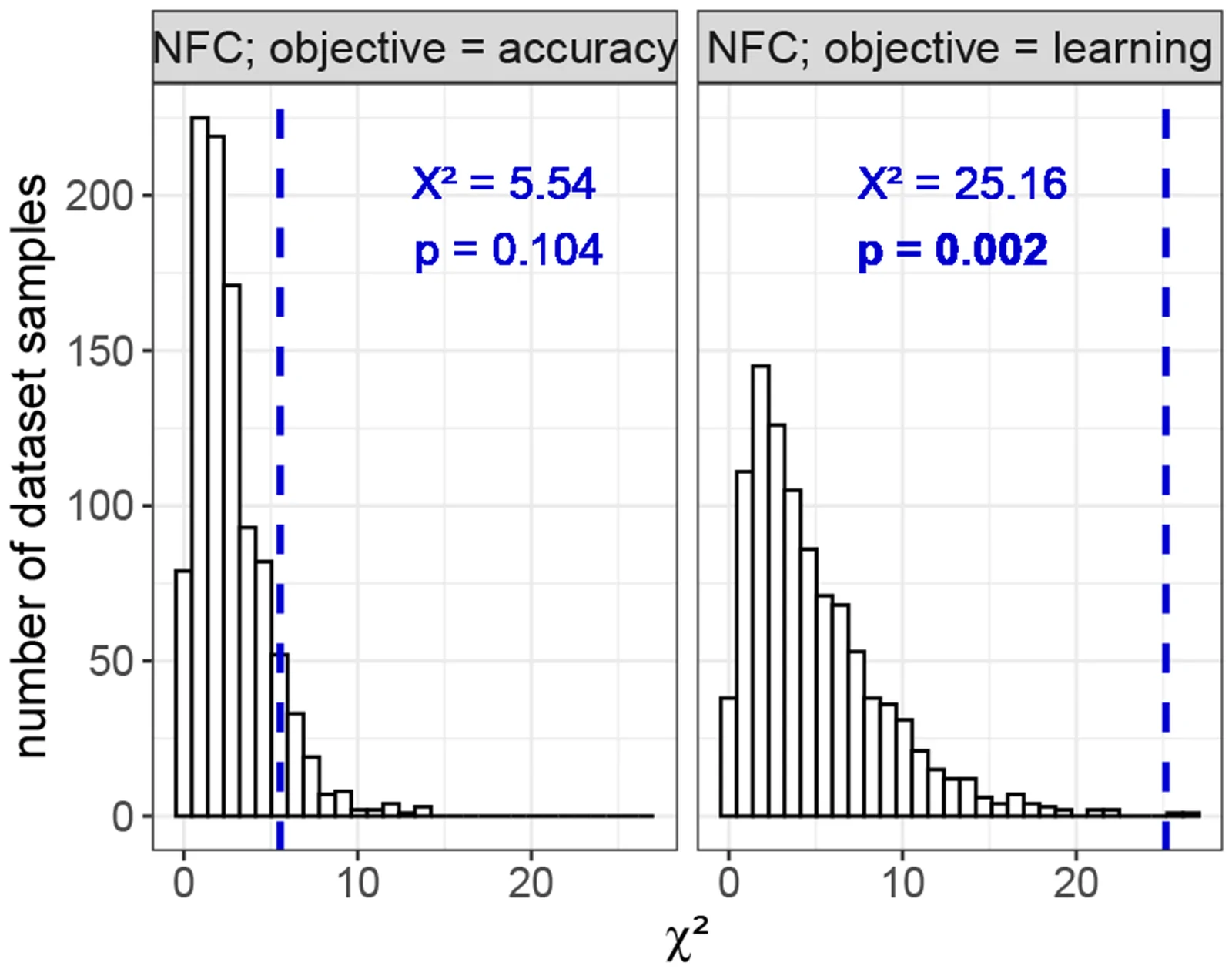
Randomization test results. Each facet depicts the *χ*^2^ distribution of 1000 datasets of random NFC assignments for the given analysis and the *χ*^2^ on the actual dataset (in blue). “NFC objective = accuracy”, for example, shows the difference of distributions of actions between the two NFC groups for the accuracy as the objective. P-value is computed as the fraction of sampled datasets in which the dataset’s *χ*^2^ exceeded the actual *χ*^2^.

To understand whether different NFC groups benefited from different types of AI assistance, we conducted a randomization test [51] using *χ*^2^ statistics. The *χ*^2^ statistic provides a way to compare the action distributions identified as optimal by AI policies for individuals categorized as having low NFC to those with high NFC. Because the analysis is conducted over the frequencies of optimal actions for different states that the RL policy has picked, the difference of distributions of actions we obtained might be due to the sample of the participants and not the variable we sought to personalize for — participant’s NFC. To understand how extreme the obtained *χ*^2^ statistics are, (i.e., whether NFC is indeed the factor that predicts the difference in the distributions of optimal actions) we conducted a randomization test by randomly assigning participants to different NFC groups (regardless of their actual level of NFC). We constructed 1000 such datasets with random NFC assignment. For each of the newly constructed datasets, we learned policies (separately for each of the two objectives) and tested the differences in the distributions of actions via *χ*^2^ (See Figure 5). We report the *p-value*, which corresponds to the fraction of the 1000 times that the *χ*^2^ statistic of a dataset with random NFC assignment exceeded the *χ*^2^ statistic of the actual dataset [51].

Supporting **H2**, our results show that the NFC group is a significant predictor of the distributions of types of assistance for learning as the objective (*χ*^2^ (3, *N* = 128) = 25.16,) *p* = .002). Specifically, actions that previous work has shown may elicit cognitive engagement [13, 42] — *explanation only* and *on demand* — were picked more often for people low in NFC than for those high in NFC. Whereas for *immediate accuracy* as the objective, we do not find any significant difference among the distributions of actions for the two NFC groups (*χ*^2^(3, *N* = 128) = 5.54, *p* = .10) , answering **RQ1**. For optimizing accuracy, actions such as *no assistance* (on states where the AI is incorrect) and *recommendation and explanation* (on states where the AI is correct) seem to be optimal for both groups.

To determine if the objective – immediate accuracy or learning – influences the distribution of AI assistance types (**RQ2**), using a *χ*^2^ test is inappropriate. This is because the policies for both objectives are derived from the same dataset (i.e., same participants), violating the independence assumption required for the test. However, we observe that when optimizing for accuracy for people high in NFC, *no assistance* is selected more often than when optimizing learning. For people low in NFC, both *no assistance* and *recommendation and explanation* are shown more often when optimizing for accuracy than when optimizing learning.

## 6 EXPERIMENT 1: EVALUATING PARTICIPANT PERFORMANCE WITH OPTIMIZED POLICIES AND SXAI

The purpose of this study was to evaluate the effectiveness of the learned policies and an SXAI baseline in improving the respective objectives – immediate decision accuracy and learning – of people with different levels of NFC interacting with them. Table 1 provides a combined summary of the findings from this experiment and from Experiment 2 (Section 7).

### 6.1 Hypotheses & Research Questions

Specifically, we hypothesized that:

- **H1b:** Each NFC group interacting with RL policies optimized for specific target objectives will exhibit superior or comparable performance on those objectives when contrasted with individuals from the same NFC group interacting with the SXAI policy.
- **H3a:** Each group of NFC who interact with an RL policy optimized for immediate accuracy will perform better on immediate tasks compared to individuals from the same NFC group interacting with a policy that selects interactions based on the human learning.
- **H3b:** Each group of NFC who interact with an RL policy optimized for human learning will perform better on distal tasks (post-intervention questions) compared to individuals from the same NFC group interacting with a policy optimized for immediate decision accuracy.

In addition, we also sought to answer the following research questions with our work:

**RQ3:** Will there be a trade-off between human learning (how much they learn) and their task enjoyment (including perceptions of effort required to perform the task)? A trade-off between effort and task enjoyment was previously observed in an AI-supported decision-making setting [13].

**RQ4:** Will greater learning of the task domain by the participants result in participants reporting higher perceived learning? Prior work indicates that perceived learning does not always reflect actual learning, particularly if environments that result in more learning require more effort [32].

### 6.2 Task and conditions

Participants were randomly assigned to one of the following conditions:

- **Baseline, *SXAI***. Participants were presented with AI recommendations and explanations in each question.
- **Accuracy**. Participants interacted with the policy optimized for immediate accuracy for their NFC group.
- **Learning**. Participants interacted with the policy optimized for learning for their NFC group.

Similar to the data collection study, participants completed a series of 33 questions, in which given a vignette about a fictitious character, they were tasked with selecting the optimal set of exercises about the character in question among two sets of exercises. Each participant was again randomly assigned to three concepts. The total 33 questions were presented in three blocks (as shown in Fig 3): *pre, intervention, post. Pre* and *post* test blocks consisted of six questions each (two per concept), and the *intervention* block consisted of 21 questions (seven per concept).

### 6.3 Experiment design

The experiment design for the evaluation study consisted of three blocks: *pre, intervention*, and *post*. Participants received no AI assistance on the *pre* and *post* blocks, which served as test blocks to measure participants’ learning. Participants interacted with one of the three policies (accuracy, learning, SXAI) during the intervention block.

The simulated AI system had an overall accuracy of 71.4%, with six out of 21 intervention questions having an underlying incorrect AI recommendation. As in the data collection study, the order of the questions was randomized for each participant and the questions in which the AI made incorrect suggestions were picked randomly. We ensured that AI had uniform accuracy across concepts: the six incorrect questions consisted of two incorrect questions per concept.

### 6.4 Procedure

The procedure was the same as for the data collection study. Participants were recruited via Prolific, a paid crowdsourcing platform and LabInTheWild.org. LabInTheWild [97] is an online platform where participants can voluntarily participate in a research study. Rather than receiving monetary compensation for participation, participants are presented with a detailed review of performance on the task and a comparison to other test-takers at the end of the study.

### 6.5 Participants

347 participants were recruited and were randomized into the three reported policies (*accuracy, learning, SXAI*). We retained 316 participants who passed an attention check at the end of the study and demonstrated a median completion time of over four seconds per question, given that the questions involved reading a vignette. (Participants’ demographics can be found in Appendix, Table 2). Each participant landing on the study from Prolific received compensation of 2.4 USD (12.72 USD/hr). Participants were assigned to policies optimized for their respective NFC groups. Categorization into *low* or *high* NFC groups depended on whether participants’ scores fell within the lower or upper 50th percentile of the NFC scores obtained from the data collection study.

### 6.6 Design and Analysis

The study was a mixed between- and within-subjects design. There was one between-subjects factor, policy choice, with three levels: 1. the *accuracy* policy, 2. the *learning* policy, 3. *SXAI*.

The within-subjects factor was the concept, with participants interacting with three out of four possible concepts assigned to them randomly.

We collected the following objective measures:

- **Immediate accuracy**: Percentage of correct answers in the *intervention* questions.
- **Learning**: Percentage of correct answers in the *post* questions (controlling for participant’s performance in *pre* questions).
- **Overreliance**: Percentage of incorrect answers in questions where the AI was incorrect and participants received any type of AI assistance.

At the end of the study, we collected the following subjective measures, all on a 5-point Likert scale from 1=Strongly disagree to 5=Strongly agree:

- **Perceived learning**: Participants responded to “*I believe I have learned about selecting exercises that are appropriate for a specific individual’s goals, constraints, and preferences*.”
- **Task enjoyment**: Participants responded to “*I enjoyed this task*.”
- **Mental demand**: Participants responded to “*I found this task mentally demanding*.”
- **Trust**: Participants responded to “*I trust this AI’s suggestions for optimal activities*.”

We used analysis of variance to analyze the impact of the different policies on both objective and subjective measures. The performance of participants on intervention questions was analyzed using mixed-effects models. The policy was modeled as a fixed effect, and the participant and concept in question as random effects. For analyzing learning, it is important to note that participants responded to only 6 post-intervention questions (2 for each concept). To ensure data conformity with a normal distribution, we employed analysis of variance on the average post-intervention question scores per participant, with policy as fixed effect and performance on pre-test questions as a covariate. Similarly, for the subjective measures, analysis of variance with policy as a fixed effect was used. We used Tukey’s HSD for post-hoc comparisons. For some of the hypotheses, our argument rests on the lack of differences between two policies. For such situations, we report 95% confidence intervals on the effect size (Cohen’s *d*). If the reported interval spans 0 and the interval is narrow, this approach helps to show that, if any difference between the treatments (i.e., policies) exists, with high probability, it is small as the effect could be zero [27, 67, 107].

For the mixed-effect models, we report degrees of freedom obtained via Kenward-Roger method [57].

### 6.7 Results

#### 6.7.1 Objective Measures.

**Fig. 6.**
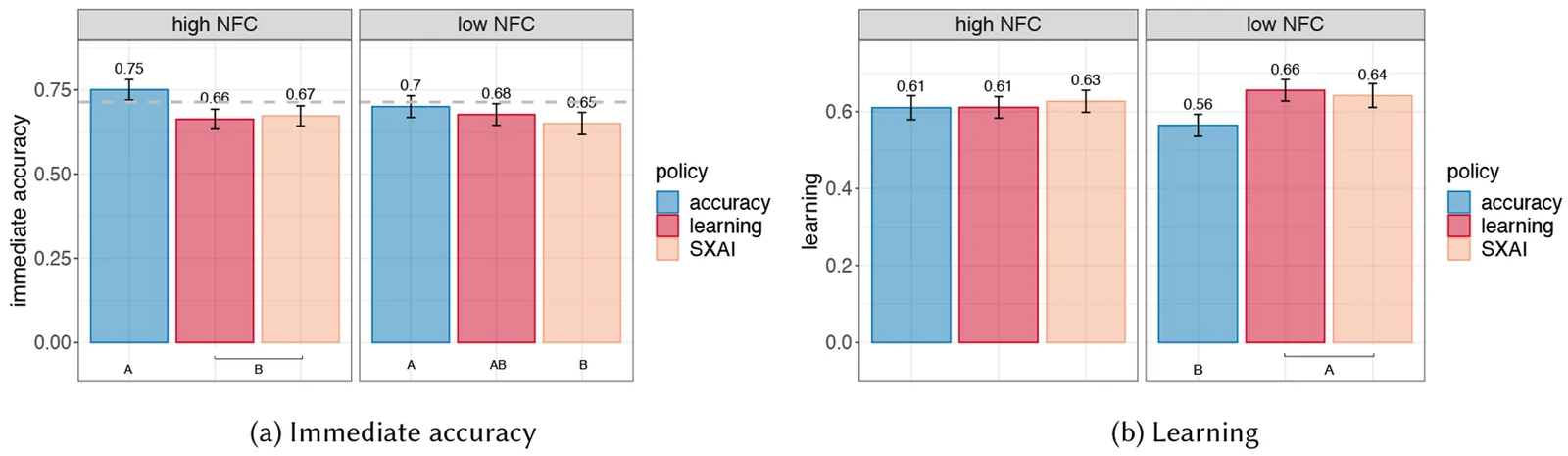
Experiment 1: Marginal means of participants interacting with the three policies: *accuracy, learning, SXAI*, on the two objectives: immediate accuracy and learning. Error bars indicate one standard error. The dashed line in (a) indicates the performance of the AI. Significance levels (if any) are depicted with letters. Conditions not connected by the same letter are significantly different.

Figure 6 summarizes the main results.

##### Comparing optimized policies to the SXAI baseline on target objectives.

We investigated how the SXAI approach—in which participants were always presented with a recommendation and explanation—compared to the policies that were tailored to the NFC group and the objective of the interaction. Our results **support H1b**. For accuracy as the objective, both NFC groups interacting with the policy optimized for immediate accuracy performed significantly better on immediate tasks than those interacting with SXAI (*low NFC*: *F*_1,89.8_ = 6.39, *p* = .01, *high NFC*: *F*_1,108.1_ = 13.97, *p* = .0003). For learning as the objective, both high and low NFC participants’ performance on distal tasks when they interacted with the *learning* policy was not significantly different from their performance with SXAI on distal tasks (*low NFC*: *F*_2,92_ = 0.06, *p* = .80, Cohen’s *d* = 0.06, 95% CI [−0.34, 0.48]; *high NFC*: *F*_2,119_ = 0.22, *p* = .64., Cohen’s *d* = −0.07, 95% CI [−0.43, 0.28]).

##### Comparing optimized policies to each other on target objectives.

First, focusing on *accuracy* as the objective, our results provide **partial support for H3a**; it was supported for participants high in NFC, but we find no support for participants low in NFC. Participants high in NFC who interacted with the *accuracy* policy performed significantly better on immediate tasks than participants high in NFC who interacted with the *learning* policy (*F*_1,111.2_ = 16.73, *p* < .0001). They also achieved complementary human-AI team accuracy (*M_human_* = 0.59, *SE_human_* = 0.03, *M_human+AI_* = 0.75, *SE_human+AI_* = 0.03; *M_AI_* = 0.714; *t*(171) = 2.75, *p* = .006). Participants low in NFC interacting with the *accuracy* policy did not perform significantly better (or worse) on immediate tasks compared to participants low in NFC who interacted with the *learning* policy (*F*_1,97.65_ = 1.59, *n.s.*).

Similarly, our results **partially support H3b**, in which the focus is on the *learning* objective. In contrast to **H3a, H3b** was supported for participants low in NFC, but we find no support for participants high in NFC. Participants low in NFC who interacted with the *learning* policy performed significantly better on distal tasks (while controlling for their initial knowledge as measured during the pre-test) than participants low in NFC who interacted with the *accuracy* policy (*F*_2,100_ = 5.23, *p* = .02). Whereas, participants high in NFC who interacted with the *learning* policy did not perform significantly better (or worse) on distal tasks compared to participants high in NFC who interacted with the *accuracy* policy (*F*_2,87_ = 0.10, *p* = *n.s.*).

#### 6.7.2 Subjective Measures.

**Fig. 7.**
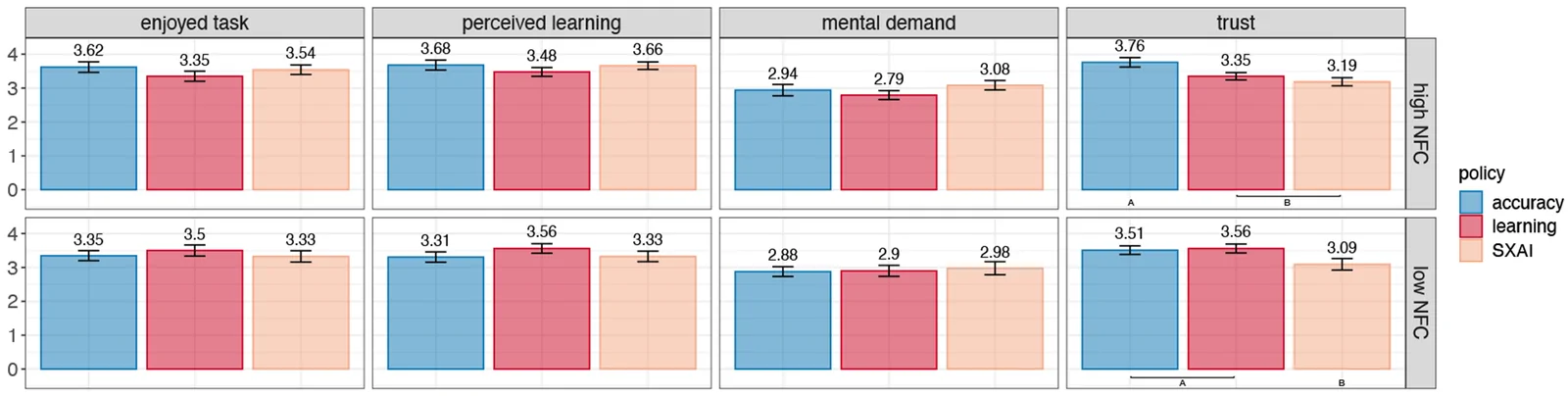
Experiment 1: Subjective measures. Error bars indicate one standard error. Significance levels (if any) are depicted with letters. Conditions not sharing the same letter are significantly different.

We investigated the effect of policy on subjective ratings for each NFC group. Results for the subjective measures are summarized in Figure 7. Each group trusted the policy that was more beneficial for them across objectives (i.e., the accuracy policy for people high in NFC, and the learning policy for people low in NFC) significantly more than the SXAI policy. Both groups also perceived that they had learned and enjoyed the task more with the policy that was more beneficial for them across objectives, but this trend was not significant.

#### 6.7.3 Objective measures vs. subjective measures.

**Fig. 8.**
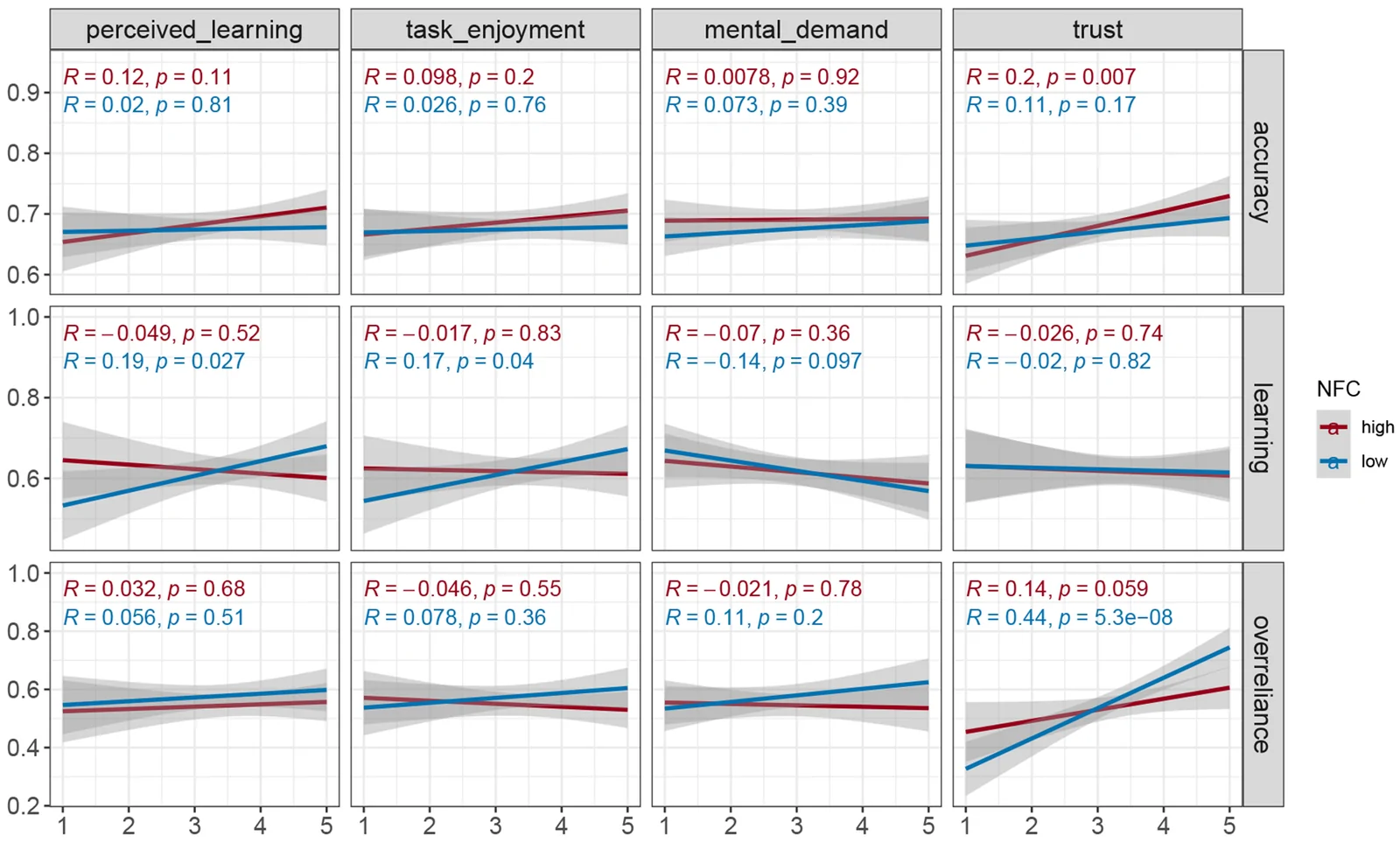
Experiment 1: Relationships between objective vs. subjective measures for the two NFC groups.

Figure 8 depicts the relationships between subjective measures and objective measures across policies for the two NFC groups. Addressing **RQ3**, we do not observe a trade-off between actual learning and task enjoyment. In fact, task enjoyment was significantly positively correlated with actual learning for people low in NFC. We observed no substantial correlation for people high in NFC. There was also a significant positive correlation between actual learning and perceived learning for people low in NFC, answering **RQ4**. Trust was significantly correlated with immediate accuracy for people high in NFC. For both NFC groups, trust was positively correlated with overreliance, albeit for people high in NFC the correlation was marginal.

### 6.8 Exploratory Analysis: Does Overreliance on AI Suggest a Lack of Cognitive Engagement?

In this section, we explore the relationship between overreliance and cognitive engagement, challenging the conventional assumption that overreliance results solely from a lack of cognitive engagement with the AI-provided information [13, 42].

Echoing past research, our computational analysis showed that for people low in NFC (i.e., those low in general cognitive motivation), the policy optimized to improve human learning included *explanation only*, an action that leads to cognitive engagement [42], as the top action (Figure 4). When evaluating participants’ performance with the policies, participants low in NFC who interacted with the learning policy indeed exhibited improved learning outcomes. This aligns with the notion that cognitive engagement is crucial for effective learning.

**Fig. 9.**
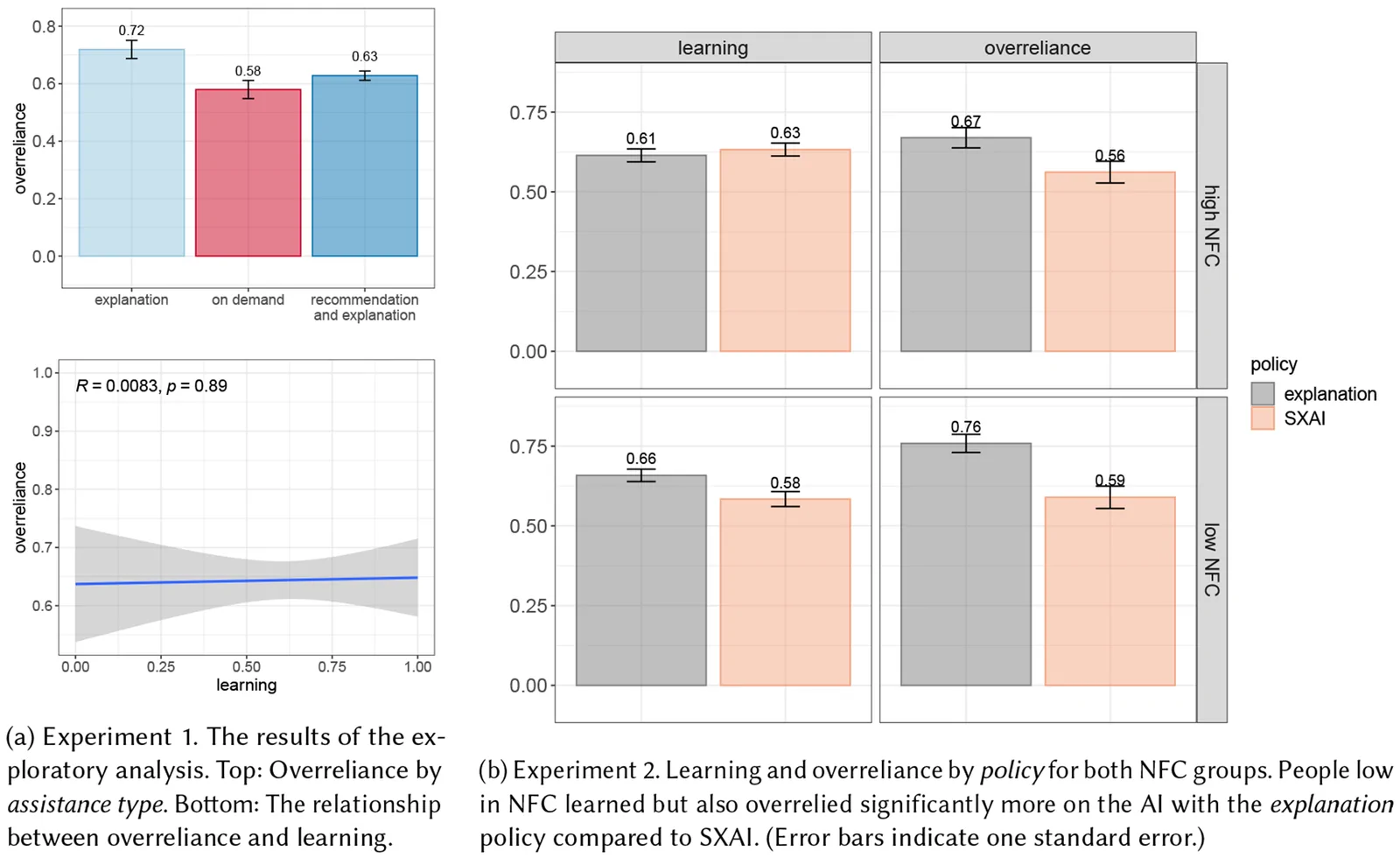
Does Overreliance on AI Suggest a Lack of Cognitive Engagement?

However, a paradox emerged when we analyzed the effects of each assistance type individually (See Figure 9a). Surprisingly, *explanation only* assistance led to significantly more overreliance compared to other assistance types, including AI recommendation and explanation. Therefore, people both overrelied when making decisions with the *explanation only* action but also demonstrated enhanced learning when this action was predominant in the policy. This finding challenges the assumption that overreliance necessarily indicates a lack of cognitive engagement.

To further investigate the relationship between these two constructs, we conducted a correlation analysis between people’s overreliance and their learning across policies (See Figure 9b). Given that incidental learning has been posited as a strong indicator of cognitive engagement, we would expect a negative correlation between learning and overreliance. However, we observed no substantial relationship between overreliance and learning (*r* = 0.008, *p* = *n.s.*, 95%*CI*[−.11, .12]). (Bootstrapped 95% confidence intervals on *r* were computed with 1000 bootstrapped datasets.) Based on this exploratory analysis, we included a new condition – a fixed policy presenting explanations only – in our subsequent experiment. We hypothesized that people will learn more but will also overrely more on AI when interacting with a fixed explanation only policy compared to another SXAI.

## 7 EXPERIMENT 2: EVALUATING PARTICIPANT PERFORMANCE WITH OPTIMIZED POLICIES AND MULTIPLE BASELINES

The second evaluation study had a primary and a secondary goal. Firstly, to further assess the effectiveness of our proposed approach — how policies optimized for different outcomes measure up against other baselines. Secondly, to test the new hypothesis about the relationship between overreliance and cognitive engagement that spurred from the exploratory analysis in Section 6.8.

To understand if it is possible to optimize participants’ accuracy and learning jointly, we introduced a *combined* policy, an RL-based policy which was optimized for both accuracy and learning, by considering both immediate and distal rewards^3^ (*λ* = 0.5, *𝛾* = 0). Alongside this, we included two additional baselines: *explanation only*, a fixed policy which provides only explanations in every decision and that prior work has shown to lead to learning without compromising accuracy [42], and a *random policy* which selects actions randomly on each question, as a mixed policy that does not account for contextual factors.

### 7.1 Hypotheses

We adjusted **H1b** to more broadly include the other baselines:

- **H1b:** Each NFC group interacting with RL policies optimized for specific target objectives will exhibit superior or comparable performance on those objectives when contrasted with individuals from the same NFC group interacting with a baseline policy.

Further, in addition to the hypotheses **H3a** & **H3b**, we hypothesized the following for the combined policy:

- **H3c:** Each group of NFC who interact with RL policies optimized for both accuracy and learning (i.e., the *combined* policy) will perform similarly on the target objectives as the policies that were optimized *solely* for the target objective.

Informed by the exploratory analysis of cognitive engagement and overreliance, we additionally constructed the following hypothesis:

- **H4:** Compared to the policy which provides AI recommendations and explanations in each decision (i.e., *SXAI*), the *explanation only* policy will lead to improved learning (as in [42]), but also increased overreliance on AI.

### 7.2 Task and conditions

We used the same task design as in experiment 1. In this experiment, participants were randomly assigned to one of the following conditions:

- **Baseline 1, *SXAI***. Same as in Experiment 1 – showing AI recommendation and explanation on each question.
- **Baseline 2, *explanation only***. In this condition, participants received *explanation only* as assistance for every question, a form of assistance previously demonstrated to enhance learning without compromising accuracy (only tested with correct AI recommendations) [42].
- **Baseline 3, *random policy***. Participants were randomly provided one of four types of assistance for each question. We included this condition as a baseline to study the effect of variability of assistance on immediate accuracy and learning when that assistance is not selected by accounting for contextual factors.
- **Accuracy** Same as in Experiment 1 – the policy optimized for immediate accuracy.
- **Learning** Same as in Experiment 1 – the policy optimized for distal benefits.
- **Combined** In this condition, participants interacted with a policy that was optimized for both immediate accuracy and learning.

### 7.3 Experiment design

The experiment design was similar to Experiment 1 but modified to include more test questions and fewer intervention questions, aiming for a more reliable assessment of learning. The *pre* and *post* blocks consisted of 9 questions each, with 3 questions per concept. The *intervention* block consisted of 15 questions (5 per concept). The simulated AI system had an overall accuracy of 73.33%, with 4 out of 15 intervention questions having an underlying incorrect AI recommendation. These 4 incorrect questions consisted of the 3 concepts, with one concept being randomly chosen to be shown incorrectly twice per participant.

### 7.4 Participants

Out of 1063 recruited participants, 964 who passed the attention check and had a median completion time exceeding 4 seconds were retained for analysis. These participants were then randomly assigned to one of the six reported conditions (See Table 2 in the Appendix for details). Each participant landing on the study from Prolific received compensation of 2.4 USD (10.02 USD/hour, with a median completion time of 14.29 minutes). As in Experiment 1, participants were assigned to policies optimized to their NFC levels (applicable for non-baseline conditions), categorized as *low* or *high* based on whether their scores were in the bottom or top 50th percentile from the data collection study.

### 7.5 Procedure, Design and Analysis

All the metrics and methods were the same as in Experiment 1.

### 7.6 Results

#### 7.6.1 Objective Measures.

**Fig. 10.**
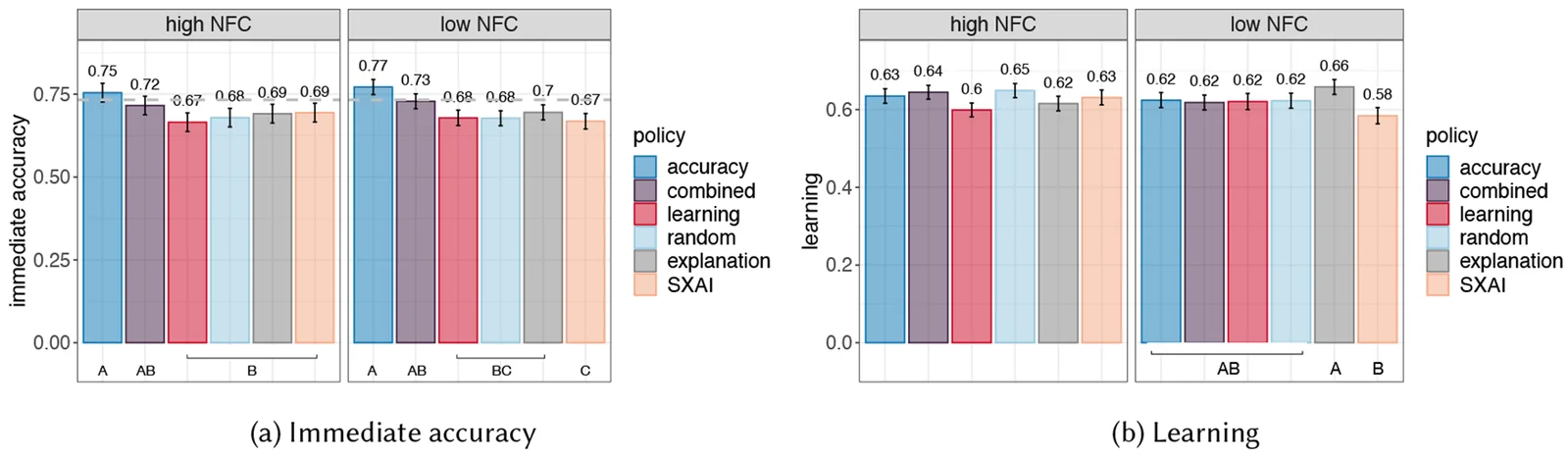
Experiment 2: Performance of participants interacting with six policies on the two objectives: accuracy and learning. Error bars indicate one standard error. The dashed line in (a) indicates the performance of the AI. Significance levels (if any) are depicted with letters. Conditions not sharing the same letter are significantly different.

Figure 10 summarizes the main results. The main effect of policy for *immediate accuracy* as the objective was significant for both groups of NFC (*low NFC*: *F*_5,425.2_ = 8.01, *p* < .0001., *high NFC*: *F*_5,518_ = 5.83, *p* < .0001). Tukey’s HSD comparisons are shown in Figure 10. Whereas, for learning as the objective the main effect was not significant for either group of NFC (*low NFC*: *F*_6,434_ = 1.23, *n.s.*, *high NFC*: *F*_6,528_ = 1.48, *n.s.*).

##### Comparing optimized policies to baselines on target objectives.

Our results **support H1b**. For accuracy as the objective, both NFC groups interacting with the *accuracy* policy performed significantly better on immediate tasks than those interacting with any of the baselines: *SXAI*, *explanation only, random* policies. Participants low in NFC achieved human-AI complementary team performance (*M*_*human*+*AI*_ = 0.77, *SE*_*human*+*AI*_ = 0.03; *M_AI_* = 0.73; *t*(89.67) = 2.52, *p* = .01). For learning as the objective, both high and low NFC participants’ performance on distal tasks with baselines was not significantly different than their performance on distal tasks with the *learning* or the *combined* policy (Pairwise comparisons with Tukey’s HSD do not detect any significant differences among the policies. For pairwise effect sizes and confidence intervals see Appendix Table 3).

##### Comparing optimized policies to each other on target objectives.

Our results *support H3a* for both NFC groups in this experiment. For each NFC group, participants who interacted with the policy that was optimized for immediate accuracy performed significantly better on immediate tasks than participants who interacted with the learning-optimized policy. In contrast to the first experiment, we **do not find support for H3b**, with learning as the objective. There were no significant differences in performance on distal tasks between participants interacting with the *learning* policy and the participants interacting with the *accuracy* policy or *combined* policy, for either NFC group.

Lending **support to H3c**, participants who interacted with the *combined* policy did not perform significantly better or worse (Tukey’s HSD – Figure 10) on either target objective compared to participants interacting with the policies that were optimized only for the accuracy objective (accuracy vs. combined — *high NFC*: Cohen’s *d* = .08, 95% CI[−.02, .19], *low NFC*: Cohen’s *d* = .10, 95% CI[−.02, .22]) or only the learning objective (combined vs. learning — *high NFC*: Cohen’s *d* = .27, 95% CI [−.02,.56]; *low NFC*: Cohen’s *d* = −.04, 95% CI [−.37,.29]).

#### 7.6.2 Subjective measures.

**Fig. 11.**
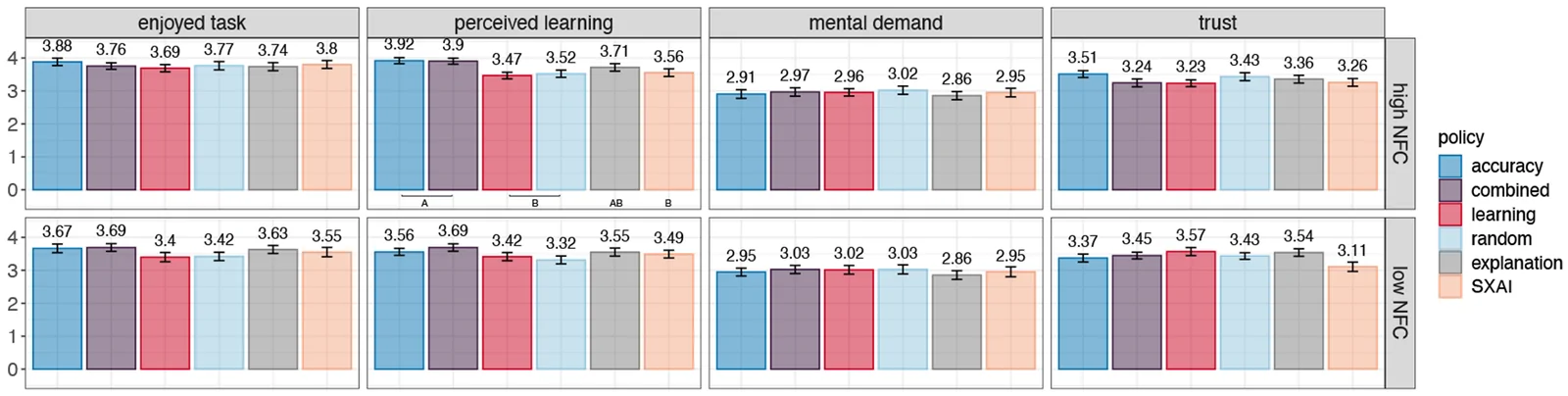
Experiment 2: Subjective measures. Error bars indicate one standard error. Significance levels (if any) are depicted with letters. Conditions not sharing the same letter are significantly different.

Results of subjective measures are summarized in Figure 11. For subjective measures, the main effect of policy was only significant for perceived learning for participants high in NFC. Participants high in NFC perceived to have learned significantly more with the *accuracy* and *combined* policy than with the *SXAI*, *random* or *learning* policies (*F*_5,528_ = 3.66, *p* = .003).

#### 7.6.3 Objective measures vs. subjective measures.

As in Experiment 1, we do not observe a trade-off between actual learning and task enjoyment (**RQ3**). We further do not observe a correlation between actual and perceived learning, which was significant only for people low in NFC in Experiment 1 (**RQ4**). (To avoid repetition, the results of objective versus subjective measures from Experiment 2 are presented in the Appendix, Figure 11.)

#### 7.6.4 Overreliance does not necessarily indicate a lack of cognitive engagement.

Our results **supported H4** only for people low in NFC. Specifically, people low in NFC who interacted with the explanation policy learned significantly more than people low in NFC who interacted with the SXAI policy (*F*_2,140_ = 3.22, *p* = .04). But they also overrelied significantly more on AI when interacting with the explanation policy compared to the SXAI policy (*F*_1,163.3_ = 4.48, *p* = .04).

## 8 DISCUSSION

In this work, we introduced offline reinforcement learning (RL) to learn decision support policies that optimize different objectives in AI-assisted decision-making. We instantiated our proposed approach with two objectives — immediate accuracy of the decisions or long-term learning by human decision-makers — while accounting for individual differences in people’s need for cognition (or NFC, which reflects a person’s intrinsic motivation to think) and other contextual factors.

### 8.1 Effectiveness of adaptive decision-making support for accuracy and learning as objectives

Our work demonstrated that AI assistance needs to be dynamic, changing in response to context, individual differences, and the specified objective. In particular, our results showed that better solutions than fixed policies like SXAI or mixed policies which do not consider these factors (i.e., random policy) can be learned for optimizing for immediate accuracy as the objective in human-AI decision-making. Both *high* and *low* NFC groups achieved significantly higher accuracy when they interacted with policies optimized for accuracy (i.e., *accuracy* or *combined* policies) compared to interacting with (i) policies that do not adapt the support based on context, or (ii) policies that were optimized solely for the *learning* objective. Notably, participants high in NFC (in Experiment 1) and participants low in NFC (in Experiment 2) achieved complementary human-AI team performance with the accuracy policy, outperforming both human and AI accuracy alone on the task. Together with recent work [79, 88], our results provide further evidence that adaptive interventions that consider contextual factors (e.g., AI’s uncertainty, the decision-maker’s competence or confidence) may be a promising approach for achieving the sought-after human-AI complementarity [8, 12].

Our work also demonstrated that learning as an objective was more challenging to optimize than immediate decision accuracy. First, we observed that people high in NFC attained similar learning outcomes regardless of the policy they interacted with in both experiments, presumably because they were already motivated to engage with information. In contrast, those low in NFC benefited more from the learning policy than the accuracy policy in Experiment 1. However, Experiment 2 showed overall no significant difference in learning outcomes between these policies. Thus, although the policies learned the best available actions to improve learning (explanation only for people low in NFC), the behavioral signal was weaker compared to accuracy.

Overall, we believe this result is driven by the fact that the design space of interaction techniques for stimulating human learning is very sparse (we are aware of only one technique — explanation only — validated in one study [42]). Thus, we argue that the results provide evidence in support adaptive support while also demonstrating the need for further development and validation of human-AI interaction techniques that would robustly support human learning. We also believe the diminished learning signal in Experiment 2, as opposed to Experiment 1, could be attributed to changes made in the experimental design. While the total number of questions remained constant across experiments, the second experiment had fewer intervention and more test questions. Although we aimed to strengthen learning measurement in Experiment 2 by increasing the number of test questions in our design update, we inadvertently limited exposure to concepts and, consequently, learning opportunities. Our research underscores the critical need to develop robust explanations and human-AI interactions that consistently enhance human learning about the domain, as well as their accuracy on the task.

Comparing objective and subjective measures in AI-assisted decision-making, a trade-off between performance and preference was previously observed [13]. Interestingly, our results showed that task enjoyment and perceived learning were positively correlated with actual learning for people low in NFC. No such correlation was observed for people high in NFC. A plausible explanation for this result may be that for low NFC participants, who are not generally inclined to engage in unnecessary cognitively-demanding activities, increased enjoyment of the task led to increased task-specific intrinsic motivation [31], and that, in turn, led to greater cognitive engagement. These findings suggest that unlike previously assumed [13], cognitive engagement may not necessarily come at the cost of negative subjective experience.

### 8.2 Evidence that RL is a promising approach for modeling human-AI decision-making

Our computational analysis of the learned policies demonstrated that RL may be a valuable approach to modeling human-AI interaction in decision-making tasks. Specifically, we found that the composition of policies differed in meaningful ways depending on both the objective and the NFC group, and differed significantly from showing a fixed type of AI assistance (e.g., *recommendation and explanation* only). The analysis of the composition of these policies revealed some insights into how learning and immediate decision accuracy were supported for individuals with different levels of NFC.

#### 8.2.1 What have we learned about the impact of need for cognition?

Our computational evaluation of the policies showed that the NFC group had a significant impact on shaping the composition of the policies optimized for learning, which necessitated cognitive engagement. Consistent with **H2**, the learning policy favored actions that prior work has shown may promote cognitive engagement, such as *explanation only* [42], more often for individuals with low NFC than for those with high NFC. Considering that individuals with low NFC do not tend to naturally cognitively engage with information, which is critical for learning, this finding is in line with the NFC construct.

**Fig. 13.**
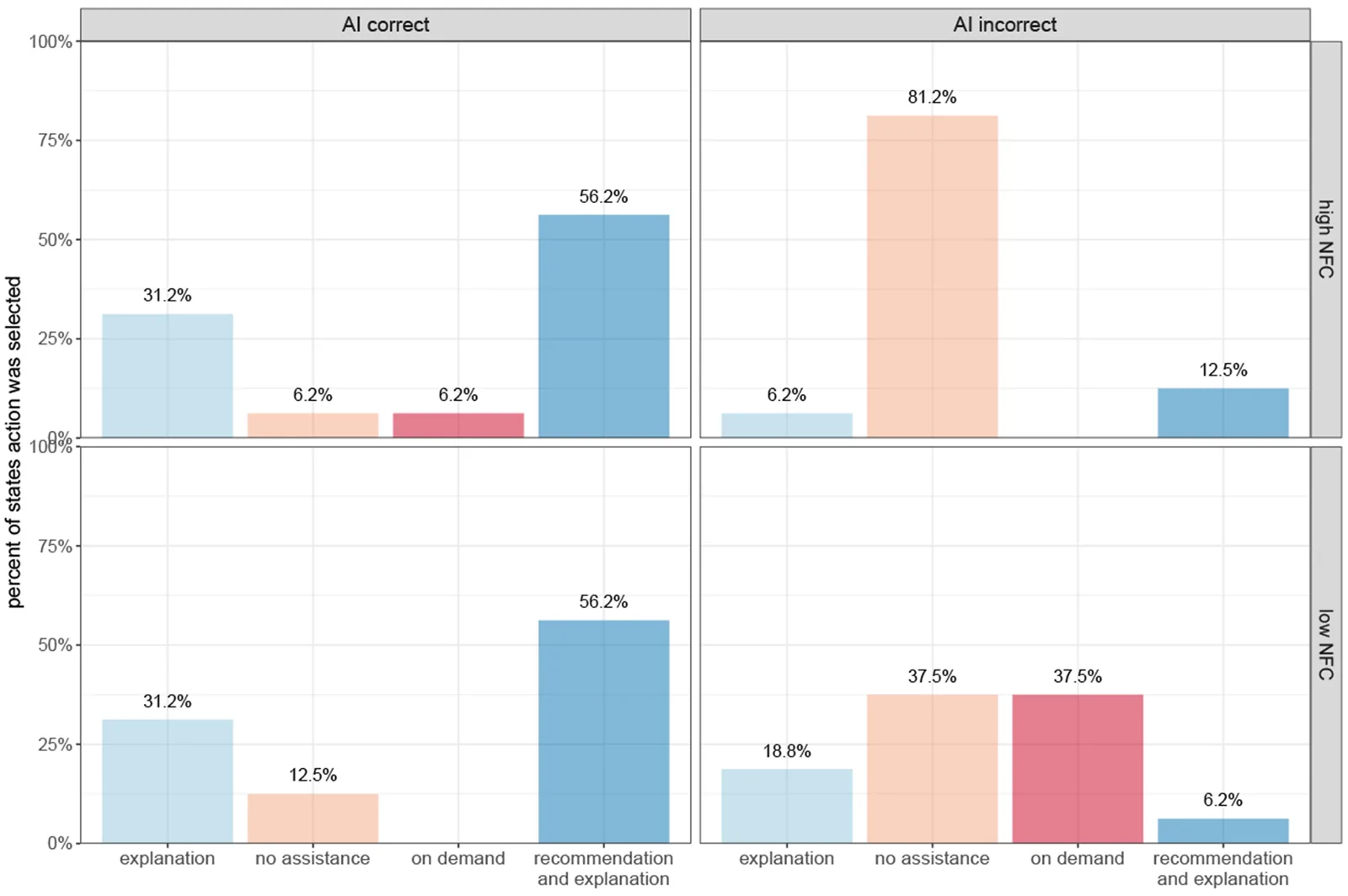
Distributions of types of AI assistance selected by the *accuracy* policy when the AI was correct and incorrect for different NFC groups.

NFC, however, was not a predictor of the policy composition for immediate decision accuracy (**RQ1**). An initial analysis indicated that the accuracy-optimized policies achieved their goal not by supporting cognitive engagement but by making reliance on the AI unlikely in those situations when the AI was incorrect. In those cases, AI assistance was often withheld. Specifically, when we examined the distributions of assistance types disaggregated by AI correctness (See Appendix, Figure 13), we observed that when the AI was accurate (in half of the state space), the policies for both groups were nearly identical, predominantly favoring actions like *recommendation and explanation*, which prior research has indicated can lead to reliance, but also *explanation* only. Whereas, in cases where the AI was incorrect, the optimal course of action for individuals with high NFC clearly leaned toward *no assistance*, whereas for individuals with low NFC, the optimal choices included both *no assistance* and *on demand*. One possible explanation for why the policy might have discovered this signal is that individuals with low NFC tend to be less inclined to actively seek out information. Consequently, they were also less inclined to click and view the AI suggestion when it was presented in an *on demand* assistance format, rendering *on demand* and *no assistance* similar interventions for them. To understand whether that was indeed what happened, we looked at how often individuals with low and high NFC clicked on the AI suggestion when it was offered in the *on demand* assistance format in the data collection study. Our findings revealed that individuals with low NFC clicked on the AI suggestion only 21% of the time, whereas those with high NFC were notably more inclined to click on the suggestion, doing so 52% of the time. This finding expands our understanding of how different individuals interact with different assistance types and it also highlights the promise of computational models to enable discovery about human-AI decision-making.

#### 8.2.2 What have we learned about different objectives?

Our inspection of the learned policies also revealed that the objective influenced the policy composition for both NFC groups. For people low in NFC, the *learning* policy favored actions that induced cognitive engagement (i.e., explanation only), whereas the *accuracy* policy favored actions that induced *reliance* when AI was correct (i.e., recommendation and explanation) and that discouraged reliance when AI was incorrect (i.e., no AI assistance or on demand). On the other hand, given that people high in NFC are already motivated to engage with information, *recommendation and explanation* was a good action for them for both *accuracy* and *learning*.

When optimizing accuracy as the objective, *no assistance* was chosen more frequently than when the objective was learning. It was an optimal action for close to half of the state space, which corresponded to the instances when the AI was incorrect. This suggests that the policy recognized not providing assistance was the best option for preventing overreliance when the AI made mistakes, corroborating findings from previous research [88]. In addition, given that *no assistance* was less often shown when optimizing learning, this also suggests that for improving learning any information is more beneficial than no information at all.

**Fig. 14.**
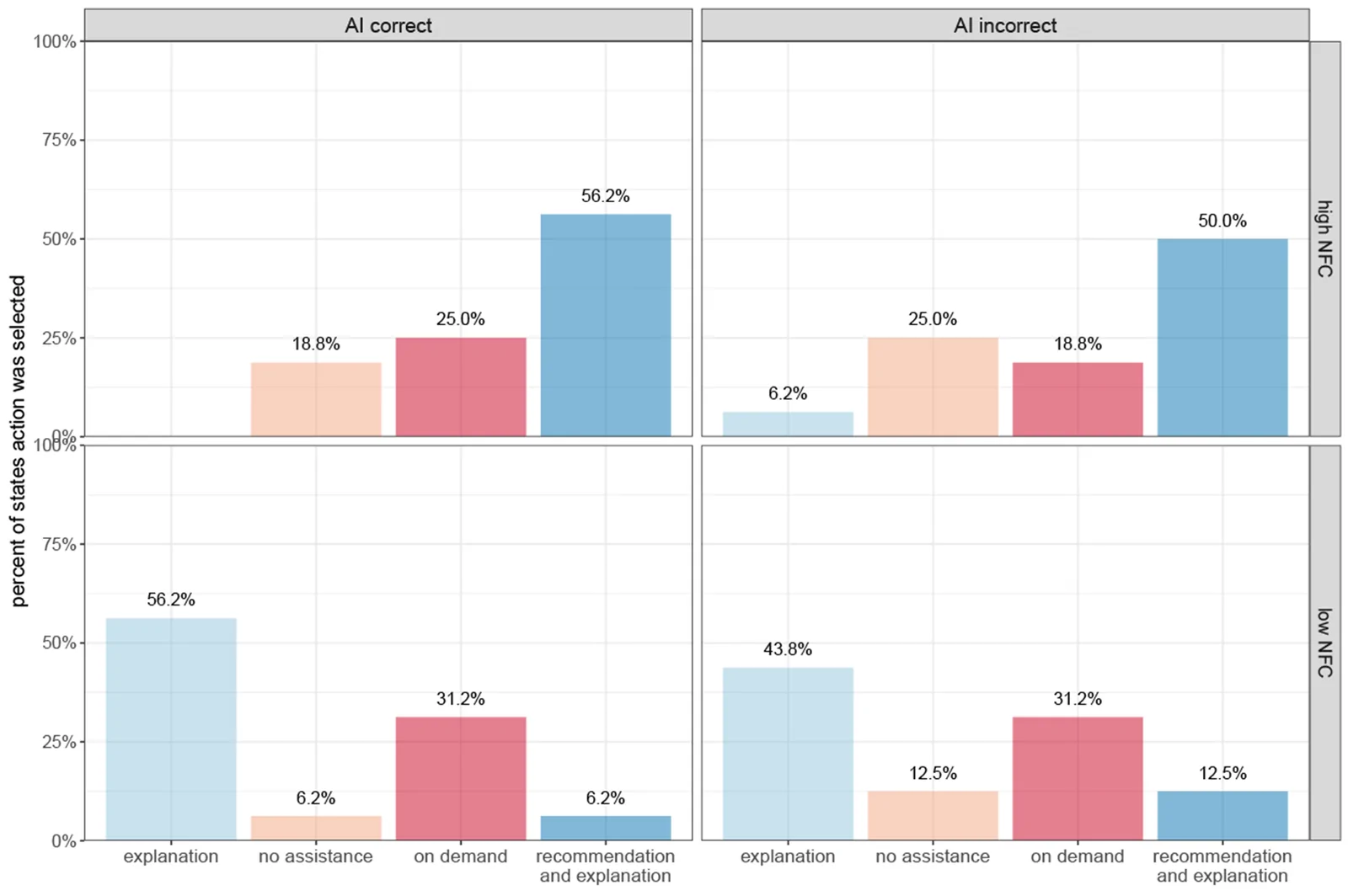
Distributions of types of AI assistance selected by the *learning* policy when the AI was correct and incorrect for different NFC groups.

Looking at the composition of the learning policy when the AI was correct vs. incorrect (See Figure 14), we do not observe any differences in policy compositions, suggesting that AI correctness was largely irrelevant to supporting learning. We believe this result is due to our explanation design, which provided factually correct, albeit irrelevant, information when the AI was incorrect. Therefore, participants may have learned useful information even though the AI suggestion was wrong.

**Fig. 15.**
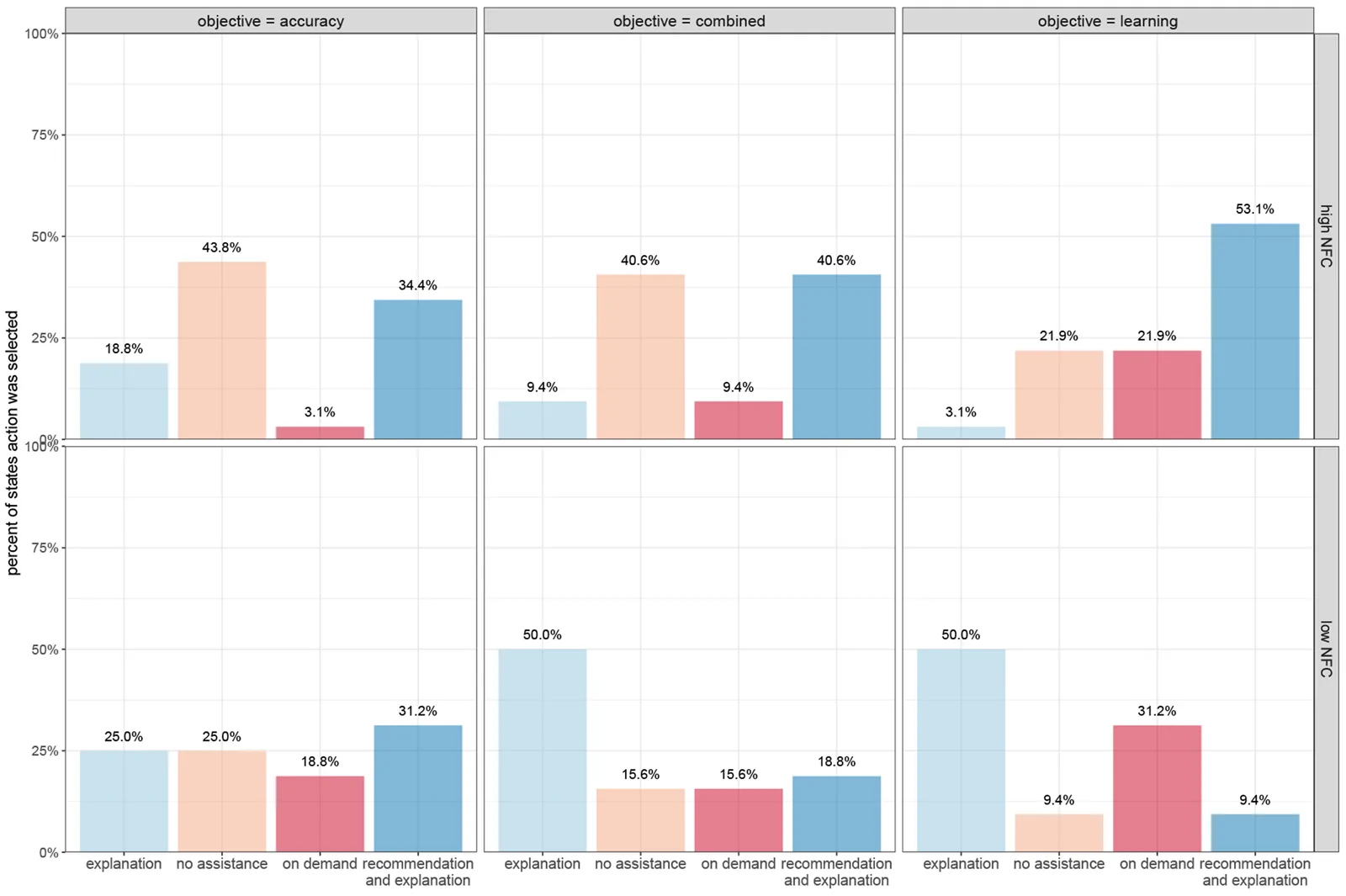
Distributions of types of AI assistance for the three policies *accuracy*, *combined (accuracy + learning*) and *learning* for different NFC groups.

Interestingly, the distributions of actions for the *combined* policy, which was optimized for both learning and accuracy were similar to the learning policy for people low in NFC, and similar to the accuracy policy for people high in NFC (See Appendix, Figure 15). For both groups, the respective policies were the better choices across objectives (in Experiment 1). This finding further suggests that the policies are optimizing for both objectives successfully, albeit the learning signal is more difficult to capture.

### 8.3 Does overreliance on AI suggest a lack of cognitive engagement?

Appropriate reliance on AI and cognitive engagement are critical constructs to consider when seeking to optimize accuracy and learning in AI-assisted decision-making. Fractured evidence from prior work suggested that there exists a relationship between cognitive engagement and overreliance on AI. Some assistance types such as *recommendation and explanation* induced reliance on AI, regardless of AI correctness [13]. But they also led to no learning about the domain [42]. Other assistance types, such as providing an explanation only without a decision recommendation, improved learning about the domain (indicating cognitive engagement) [42], but their effect on (over)reliance was not evaluated. The underlying explanation for the difference in learning outcomes (and the possible difference in reliance) for the two assistance types was related to the effect they had on cognitive engagement. It was suggested that cognitive engagement explained both learning and overreliance. Assistance types that induce cognitive engagement lead to learning *and* should reduce overreliance on AI [42].

Our results paint a more complex picture. Drawing on exploratory analysis (§ 6.8) and the subsequent finding (§ 7) that *explanation only* improved learning compared to *SXAI*, we believe that cognitive engagement does indeed improve learning. But contrary to the field’s tentative understanding, lack of cognitive engagement may not be the sole predictor of overreliance. The first piece of evidence that supports this hypothesis is that in our exploratory analysis, we find no substantial correlation between overreliance and learning across policies. Also, when analyzing the effect of individual assistance types on overreliance, we observed that *explanation only* led to significant overreliance compared to other assistance types, but people interacting with policies where *explanation only* was the predominant assistance type also exhibited improved learning. Decisively, Experiment 2 revealed that people low in NFC both learned more *and* overrelied more on AI when interacting with *explanation only* policy compared to SXAI.

Together our findings demonstrate that the relationship between overreliance and cognitive engagement is multifaceted and not as straightforward as previously assumed. While cognitive engagement remains a crucial aspect of learning, overreliance on AI may stem from various factors. It may be a result of superficial engagement with AI-provided assistance, but it may also be due to people engaging with the provided assistance *and* finding the AI’s (misleading) explanation plausible. Understanding these nuances is essential for developing effective AI systems that enhance learning and promote appropriate reliance.

### 8.4 Generalizability & Limitations

We believe using offline RL to dynamically choose whether and how AI should support human decision-makers has the potential to optimize various human-centric objectives effectively in AI-supported decision-making, although we focused only on accuracy and learning in this work. Success depends, however, on intelligently designing RL components (state space, action space, rewards) based on each specific objective. For example, to enhance collaboration and relatedness in the workplace [30] as an objective, interventions may even advise decision-makers to seek insights from a more experienced colleague, or to form a team to tackle complex decision-making scenarios that an individual is uncertain about. While a large design space of interventions can be explored, crafting RL components can be especially challenging for human-centric objectives other than accuracy for which empirical evidence about effective assistance types and relevant factors is still scarce in AI-assisted decision-making literature, as we observed for learning as an objective.

Although we used offline RL in this work (we ran an initial study with an exploratory policy, and then optimized for other policies), we could have run an online RL algorithm instead (where we learn optimal policies during a study). However, this would require a lot of data to converge, could be risky (by taking exploratory unsafe actions in real-time in the environment) and computationally expensive (especially when decisions are required in time-constrained settings). We used Q-learning as our offline off-policy learning algorithm: other algorithms are possible, but Q-learning was sufficient in our relatively simple (discrete) state and action-space.

Further, our state space included *h_t_*, representing an individual’s accuracy on a specific concept up to time step *t*. In our experimental setting, this variable could be inferred in real-time, as we had access to ground truth—i.e., the moment a participant made a decision, we could determine whether it was correct. In real-world scenarios, however, the accuracy signal may be delayed; for example, a clinician might only learn whether a diagnosis was correct after additional testing or patient outcomes are observed. This delay would make *h_t_* slower to update, though still a valuable signal eventually. Importantly, the fact that rewards (in this case the accuracy signal) in real-world settings are often sparse and delayed highlights the strength and suitability of RL as a framework for modeling human–AI decision-making.

Our work has several limitations. Our findings are based on studies conducted in a non-critical domain, and with crowds. However, since previous research in AI-assisted decision-making has demonstrated that experts exhibit behavior akin to that of crowds when utilizing AI for decision-making [19, 34, 45], we may expect our findings to generalize to real contexts with experts making decisions in critical domains. Moreover, the type of task we employed supports this potential for generalization. The cognitive demands of exercise prescription closely mirror those of clinical treatment selection. Both require the decision-maker to match an intervention to an individual’s unique circumstances. In medical contexts, clinicians must account for a range of considerations, such as patient health status, preferences, side effects, and practical constraints. Likewise, choosing an appropriate exercise involves balancing goals, limitations, and preferences, engaging similar forms of judgment and reasoning. Importantly, our study represents an initial step toward assessing whether adaptive AI-powered decision support can enhance human decision-making. Further research is needed to identify the domains and conditions under which such support proves most effective.

Using only one task is also a limitation. To isolate task effects and gain a comprehensive understanding of our approach, we conducted multiple experiments. The existing body of work in AI-assisted decision making (Section 2.1), encompassing various tasks such as disease diagnosis, recidivism, and sentiment analysis, collectively demonstrates that the presence and type of AI advice influence human behavior more significantly than the nature of the task itself. Therefore, we believe our approach is likely to generalize beyond this single setting.

Our results may have also partially been driven by the explanation choice, which was explicitly designed to enable learning of factual information about the task domain (e.g., “swimming supports muscle building”). As is the case with accuracy [8, 12, 24], we believe that different explanation designs may have different impacts on learning. For instance, a recent study [15] introduces contrastive explanations that address common human misconceptions, demonstrating significantly greater improvements in learning compared to conventional AI explanations that do not account for users’ reasoning patterns. Such approaches offer a promising action to include in the action space for more robust support of user learning than the explanation design used in our study. Another consideration is that our explanation design included a factual statement about one of the exercises, which may have been interpreted as a decision recommendation. This could have contributed to the overreliance observed in the explanation-only condition. Explanation designs that more subtly guide decision-making, without explicitly signaling one option, may help mitigate such overreliance.

Both the task-level AI responsible for generating recommendations and explanations, and the interaction-level AI responsible for adaptively selecting support, could in principle have been implemented using LLMs. We controlled the task-level AI rather than using an LLM to generate recommendations and explanations, as this would introduce uncontrolled variability in explanation quality and linguistic choices that would confound our ability to attribute behavioral effects to the interaction policy itself [59, 63]. Controlling the task-level AI is standard practice in foundational human-AI decision-making research, and does not imply that LLM-powered explanations are infeasible or are always undesirable—indeed, we have explored such generations for this exercise prescription task in other work [15]. Similarly, LLMs could in principle also be used for interaction selection, as an alternative to RL. However, specifying interaction objectives, modeling the evolving state of the human-AI dyad, and optimizing for long-term outcomes is non-trivial in that setting, and such an approach would constitute a different design paradigm requiring its own methodological justification. How best to learn adaptive interaction policies—whether via RL, LLMs, or other approaches—and what tradeoffs each entails in terms of interpretability, data requirements, and alignment with long-term human-centric objectives, remains an important question for future work.

### 8.5 Ethical Considerations

The status quo of deploying AI decision support systems without understanding their impact on people — their skills, enjoyment, autonomy, collaboration with others, and work meaning — is implicitly a value-laden decision. While our approach introduces a novel research direction focused on making such values explicit by enabling optimization of human-centric objectives in AI-assisted decision-making, it also surfaces ethical issues that must be addressed. One critical aspect is deploying technologies in a worker-centered way and ensuring that individuals engaging with these systems possess the autonomy to shape the influence these technologies have on them and their work environment [2, 4, 55]. Therefore, it is critical for the system objectives to be determined and inputted by the users themselves, rather than being paternalistically imposed by those in power (e.g., managers). Further, personalization variables in the algorithm based on factors like skill level or motivation to think, may be used for nefarious purposes and could lead to unfair treatment or discrimination in workplace settings. It is essential to safeguard user privacy of such variables in the system design and allow only individuals interacting with the system to decide what variables can be tracked and utilized for personalization [4, 6].

## 9 CONCLUSION & FUTURE DIRECTIONS: HUMAN-CENTERED AI FOR DECISION SUPPORT

In this work, we introduced adaptive support as a method to optimize human-AI outcomes, while demonstrating its effectiveness in improving human-AI accuracy and its potential for improving human skills on the task as well. We believe that the present design and development of AI decision aids has been narrowly fixated on improving only the accuracy of the decisions, largely neglecting other human-centric objectives that the decision-maker may value and find motivating in their work (e.g., skill improvement, autonomy, social belonging [31]). Yet, human-AI dyads form sociotechnical systems that produce both tangible (e.g., decisions) and socio-psychological outcomes [98, 113]. Extensive research in work design has demonstrated that human-centric socio-psychological outcomes, such as competence and autonomy, are vital mediators of motivation, performance, and overall well-being in the workplace [30, 48, 84, 91, 92]. Considering this evidence, we advocate for a broader perspective among HCI, CSCW, and machine learning fields when designing AI for decision-support. Researchers and practitioners should consider how to design and implement AI decision aids that enhance not only task performance but also the well-being and satisfaction of the individuals using them. We initiated this endeavour by proposing adaptive support with offline RL as one promising approach to optimize human-centric objectives and believe there is ample opportunity for future innovation in this important area.

## Figures and Tables

**Fig. 1.**
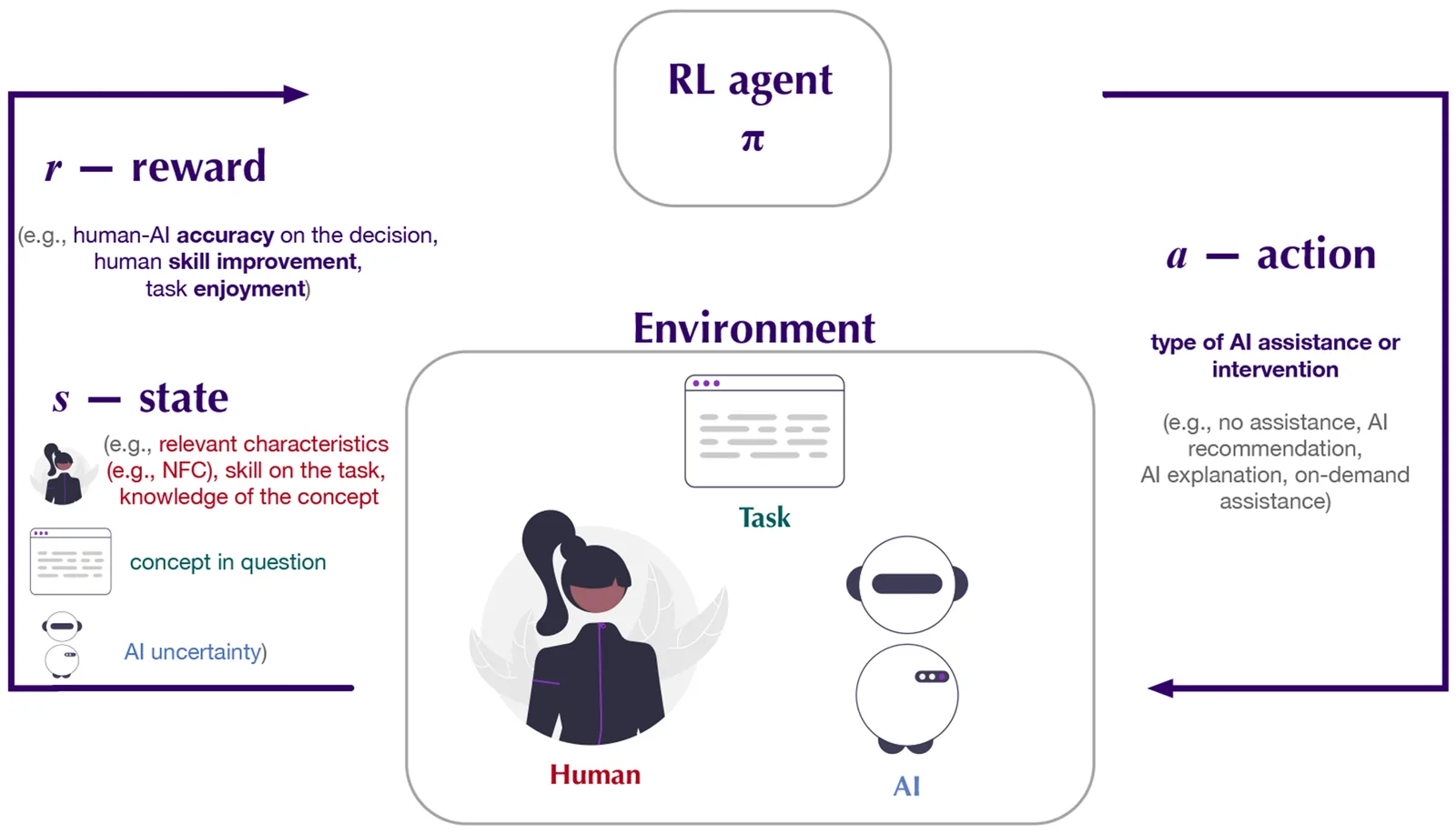
A simplified overview of the proposed method. Providing adaptive decision support through reinforcement learning for optimizing decision accuracy and human learning about the task while accounting for human-centric and other contextual factors in human-AI decision-making.

**Table 1.** Summary of results for the main hypotheses tested in Experiment 1 & 2. For a hypothesis: ✓ indicates support, ✓* partial support, and ✗ no support.

		Experiment 1 (N = 316)	Experiment 2 (N = 964)
**Hypotheses**	**target objective**	baseline: SXAI optimized: accuracy, learning	baselines: SXAI, explanation, random optimized: accuracy, combined, learning
**H1b:** Policies optimized for a target objective will result in human performance on that objective that is either equal to or better than baseline policies.	**accuracy**	✓Supported for both NFC groups accuracy > baseline (SXAI)	✓Supported for both NFC groups accuracy > all baselines combined ≥ all baselines
	**learning**	✓Supported for both NFC groups learning ≥ baseline (SXAI)	✓Supported for both NFC groups learning ≥ all baselines combined ≥ all baselines
**H3a & H3b:** Policies optimized for a target objective will result in better human performance on that objective than policies optimized for another objective.	**accuracy**	✓* Supported for people high in NFC accuracy > learning	✓Supported for both NFC groups accuracy > learning
	**learning**	✓* Supported for people low in NFC learning > accuracy	✗ Not supported learning not significantly different from accuracy

**Fig. 12.**
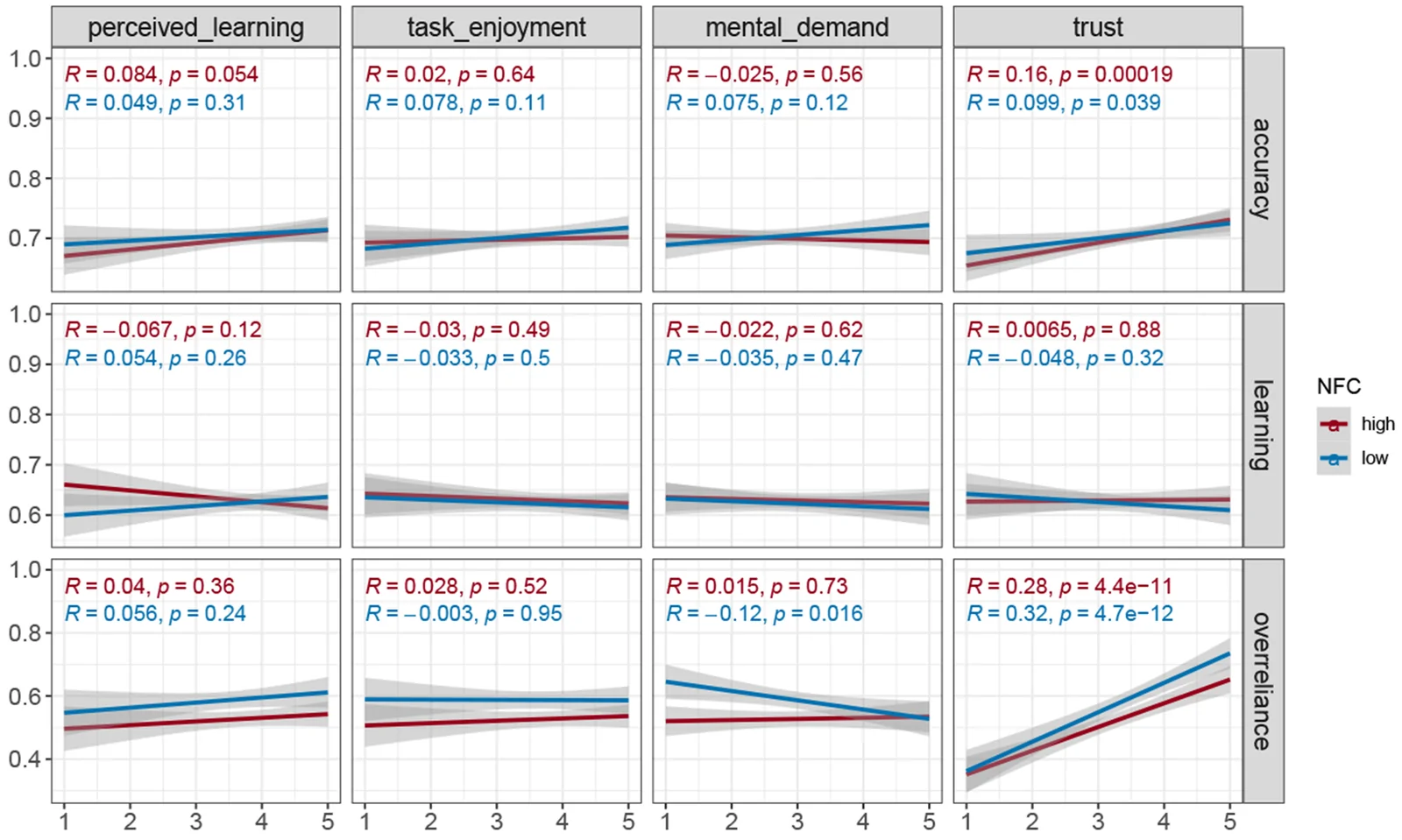
Experiment 2: Relationships between subjective measures and objective measures

**Fig. 16.**
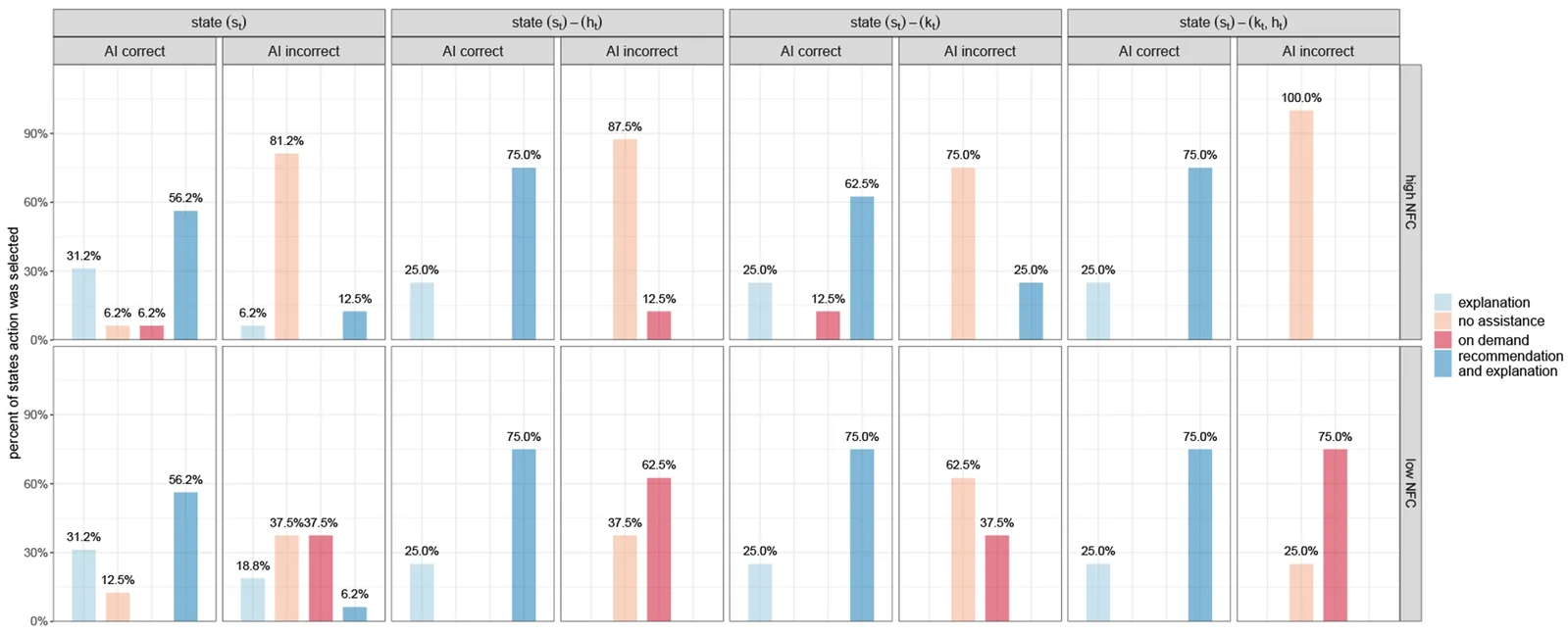
Distributions of AI assistance types under the **accuracy** policy learned with different state representations. The baseline, *state* (*s_t_*), refers to the policy learned using the full original state representation. The ablation *state* (*s_t_* – *h_t_*) excludes the decision-maker’s performance history on prior questions related to the concept. *State* (*s_t_* – *k_t_*) excludes the decision-maker’s initial task knowledge, as measured by their responses to the first questions. Finally, *state* (*s_t_* – (*k_t_*, *h_t_*)) excludes both performance history and initial knowledge.

**Fig. 17.**
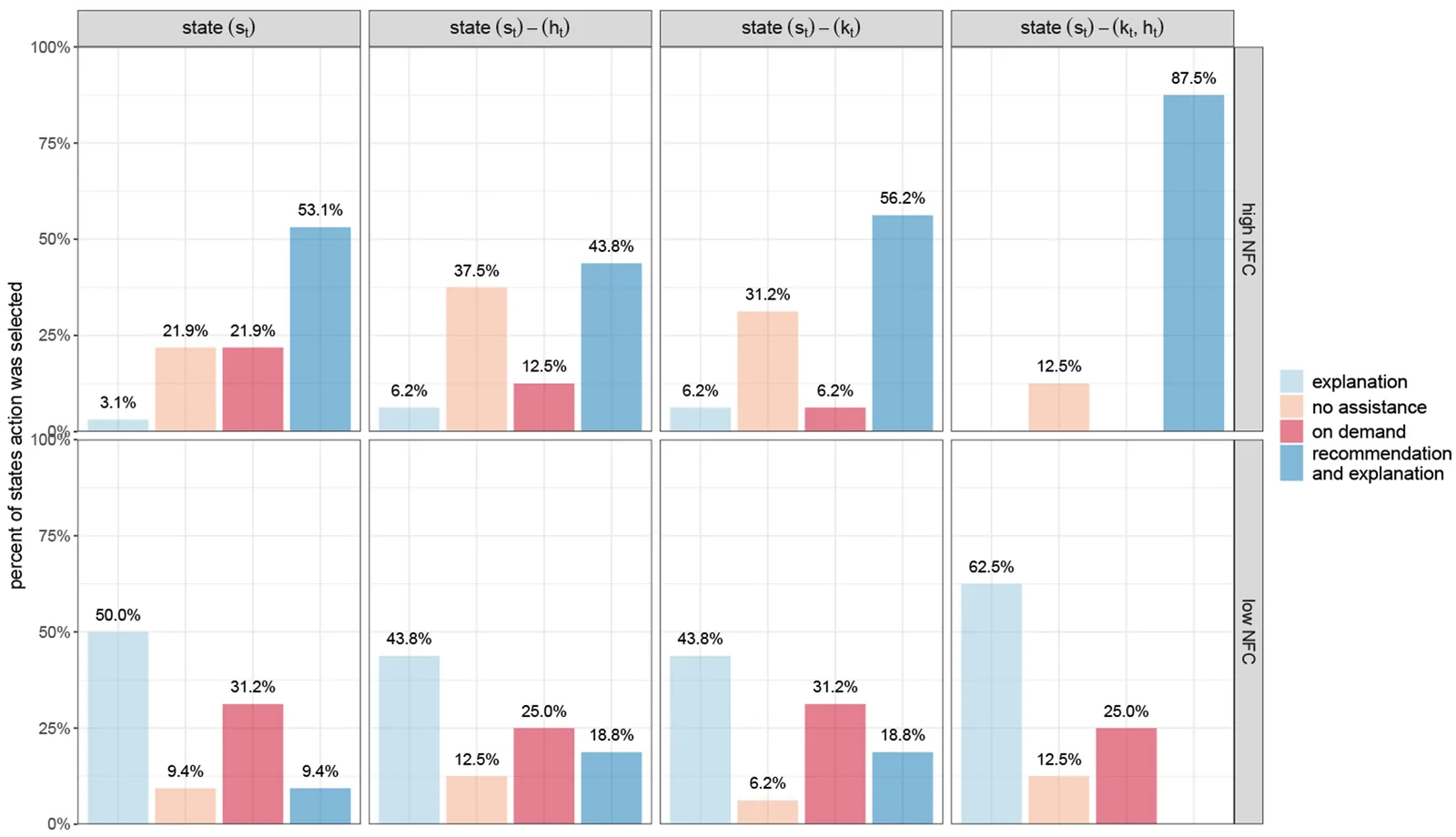
Distributions of AI assistance types under the **learning** policy learned with different state representations. The baseline, *state* (*s_t_*), refers to the policy learned using the full original state representation.

## CCS Concepts:

• Human-centered computing → Systems and tools for interaction design; Interaction paradigms; Empirical studies in HCI.

## Footnotes

1 This approach to assigning reward is an implementation choice we made for this paper and should not be construed as the only or optimal method for such assignment.

2 This sample size only reflects binary treatments and focuses on individual treatment effects, not the policies that can be learned from RL algorithms with the given data.

3 We set *𝛾* = 0, equally weighing immediate and distal benefits with *λ*. Note that, in our setup, any *𝛾* > 0 would further weigh learning since future rewards might include accuracy attained with *no AI* support in the intervention phase.

## A APPENDIX

### A.1 Participants’ demographics

**Table 2.** Participants’ demographics and assignment to conditions in the respective study.

	Data Collection Study	Experiment 1	Experiment 2
**n**	142	316	964
**Data collection**	June 2023	July-August 2023	November 2023
**Source**	Prolific: 142	Prolific: 281 LabintheWild: 35	Prolific: 952 LabintheWild: 12
**Age**	M=38.09, SD=13.59	M=38.84, SD=14.9	M=42.33, SD=14.47
**Gender**	women: 79 men: 63	women: 170 men: 135 non-binary: 7 not responded: 4	women: 493 men: 438 non-binary: 26 not responded: 7
**Conditions (high NFC, low NFC)**	exploratory policy: 142 (high: 74, low: 68)	SXAI: 102 (high: 59, low: 43) accuracy: 99 (high: 50, low: 49) learning: 115 (high: 63, low: 52)	SXAI: 146 (high: 81, low: 65) accuracy: 161 (high: 86, low: 75) learning: 159 (high: 94, low: 65) combined: 172 (high: 94, low: 78) explanation: 160 (high: 84, low: 76) random: 166 (high: 90, low: 76)

### A.4 Ablation Analyses of Optimized Policies

Our state space consisted of the participant’s NFC (*nfc_t_*), the concept in question (*c_t_*), the AI’s uncertainty (*u_t_*), the participant’s performance history on the concept (*h_t_*), and the participant’s initial knowledge of the task (*k_t_*), measured by three questions without AI assistance at the beginning of the study. Computational evaluation and policy inspection revealed that NFC and AI’s uncertainty are key factors requiring adaptation. To assess the contribution of the remaining variables — performance history (*h_t_*) and initial knowledge (*k_t_*) — to the state representation, we conducted ablation analyses on the learned policies. Specifically, we trained three additional sets of policies (for both accuracy and learning) with modified state representations: (1) excluding *h_t_*, (2) excluding *k_t_*, and (3) excluding both *h_t_* and *k_t_*.

#### *Accuracy-Optimized* Policies.

Figure 16 shows the action distributions by AI correctness for accuracy-optimized policies learned with different state representations. As the state representation is reduced (via ablations), the learned policies become more conservative and uniform, especially when the AI is incorrect. Removing performance history or initial knowledge eliminates nuance and personalization, which leads to heavier emphasis on *no assistance* and *recommendation with explanation*. This suggests that both *h_t_* and *k_t_* play important roles in fine-tuning adaptive assistance based on user knowledge and their concept-specific performance history.

#### *Learning-Optimized* Policies.

Figure 17 depicts action distributions for learning-optimized policies learned with different state representations. Similar to accuracy-optimized policies, removing performance history and initial knowledge eliminates nuance and personalization of the policies. In addition, the distributions suggest that including either performance history (*h_t_*) or initial knowledge (*k_t_*) in the state representation results in similar policy behaviors under learning-optimized objectives. Congruent with the constructs they capture (i.e., how likely the participant is to perform well), this similarity suggests that either *h_t_* or *k_t_* alone may be useful for personalization for learning-optimized policies to approximate the full-state policy’s behavior (e.g., when it is not feasible to include both). However, removing both variables leads to significantly different (and less nuanced) policies, highlighting their complementary importance when used together.

Finally, although we interpreted the behaviors learned by policies with different state representations here, determining the effect of which variables significantly influences downstream outcomes remains difficult without human-subject experiments, which are too costly to perform for each representation.

### A.5 Experiment 2: Effect sizes for non-significant differences

**Table 3.** Effect sizes for H1b in Experiment 2.

	high NFC	low NFC

comparison	Cohen’s d 95% CI	Cohen’s d 95% CI

learning-SXAI	−.18 [−.48, .11]	.19 [−.15, .54]
combined-SXAI	.08 [−.22, .38]	.17 [−.16, .51]
learning-random	−.29 [−.58, .006]	−.02 [−.36, .31]
combined-random	−.03 [−.32, .26]	−.05 [−.37, .27]
explanation-learning	.07 [−.22, .37]	.22 [−.12, .55]
combined-explanation	.17 [−.13, .47]	−.24 [−.56, .08]

### A.6 Coverage Analysis of the Exploratory Policy

To assess the adequacy of our offline dataset for tabular Q-learning, we conducted a coverage analysis of the state and state-action spaces. Our setting consists of 64 discrete states and 4 actions (256 state-action pairs). At the state level, we observed a mean of 50.7 transitions per state (median 40.5). At the state-action level, we observed a mean of 12.7 observations per pair (median 9), with 93.4% of pairs having at least 3 observations. The cells with fewer observations correspond specifically to no-AI state-action pairs—a known and intentional property of our quasi-uniform exploratory policy, which sampled no-AI less frequently than other assistance types. These figures indicate that the vast majority of state-action pairs received multiple observations, and that cells which received fewer observations were limited to a small and interpretable subset of the space.
